# Anti-kindling Induced by Two-Stage Coordinated Reset Stimulation with Weak Onset Intensity

**DOI:** 10.3389/fncom.2016.00044

**Published:** 2016-05-17

**Authors:** Magteld Zeitler, Peter A. Tass

**Affiliations:** ^1^Research Center Jülich, Institute of Neuroscience and Medicine, Neuromodulation (INM-7)Jülich, Germany; ^2^Department of Neurosurgery, Stanford UniversityStanford, CA, USA; ^3^Department of Neuromodulation, University of CologneCologne, Germany

**Keywords:** coordinated reset, two-stage CR stimulation with weak onset, desynchronization, spike timing-dependent plasticity, anti-kindling

## Abstract

Abnormal neuronal synchrony plays an important role in a number of brain diseases. To specifically counteract abnormal neuronal synchrony by desynchronization, Coordinated Reset (CR) stimulation, a spatiotemporally patterned stimulation technique, was designed with computational means. In neuronal networks with spike timing–dependent plasticity CR stimulation causes a decrease of synaptic weights and finally anti-kindling, i.e., unlearning of abnormally strong synaptic connectivity and abnormal neuronal synchrony. Long-lasting desynchronizing aftereffects of CR stimulation have been verified in pre-clinical and clinical proof of concept studies. In general, for different neuromodulation approaches, both invasive and non-invasive, it is desirable to enable effective stimulation at reduced stimulation intensities, thereby avoiding side effects. For the first time, we here present a two-stage CR stimulation protocol, where two qualitatively different types of CR stimulation are delivered one after another, and the first stage comes at a particularly weak stimulation intensity. Numerical simulations show that a two-stage CR stimulation can induce the same degree of anti-kindling as a single-stage CR stimulation with intermediate stimulation intensity. This stimulation approach might be clinically beneficial in patients suffering from brain diseases characterized by abnormal neuronal synchrony where a first treatment stage should be performed at particularly weak stimulation intensities in order to avoid side effects. This might, e.g., be relevant in the context of acoustic CR stimulation in tinnitus patients with hyperacusis or in the case of electrical deep brain CR stimulation with sub-optimally positioned leads or side effects caused by stimulation of the target itself. We discuss how to apply our method in first in man and proof of concept studies.

## Introduction

Several brain disorders are characterized by abnormally strong neuronal synchronized activity (Uhlhaas and Singer, 2006). Examples of such neuronal disorders are epilepsy (Wong et al., 1986; Schomer and Lopes da Silva, 2011), Parkinson's disease (Lenz et al., 1994; Nini et al., 1995; Hammond et al., 2007), and tinnitus (Ochi and Eggermont, 1997; Llinas et al., 1999; Weisz et al., 2005; Eggermont and Tass, 2015). Neuronal dynamics essentially depends on the neurons' connectivity patterns (Sporns, 2011). Furthermore, the mechanism by which the neurons interact crucially determines whether and how they synchronize. For instance, as shown computationally the synchronization behavior of neuronal networks may strongly differ depending on whether neurons are coupled via gap-junctions or synapses (Belykh et al., 2005; Belykh and Hasler, 2011). Accordingly, computational studies were devoted to the role of gap junctions in the emergence of epileptic seizures (Volman et al., 2011) or on the impact of the interplay between gap junctions and delayed inhibitory synaptic coupling on the emergence of different patterns of neuronal synchrony (Guo et al., 2012).

Connectivity and related function may undergo plastic changes that are not restricted to periods early in life (Hübener and Bonhoeffer, 2014). The timing pattern of neuronal activity may strongly shape the strength of neuronal connections (Hebb, 1949; Bliss and Lomo, 1973). Spike timing-dependent plasticity (STDP) is a fundamental mechanism by which neurons adapt the strength of their synapses to the relative timing of their action potentials (Gerstner et al., 1996; Markram et al., 1997; Bi and Poo, 1998; Feldman, 2000). A number of computational studies addressed the impact of activity dependent coupling/synaptic strength on the collective dynamics (Seliger et al., 2002; Tass and Majtanik, 2006; Maistrenko et al., 2007; Masuda and Kori, 2007; Ren and Zhao, 2007; Aoki and Aoyagi, 2011; Belykh and Hasler, 2011; Volman et al., 2011; Bayati and Valizadeh, 2012; Guo et al., 2012; Knoblauch et al., 2012; Popovych et al., 2013; Zhang et al., 2013). In the presence of STDP a variety of different dynamical phenomena and regimes emerge, such as complex clustering phenomena, see e.g., Maistrenko et al. (2007) and Belykh and Hasler (2011), and the emergence of traveling waves, see e.g., Zhang et al. (2013). Neuronal networks as well as oscillator networks with STDP typically display multistability. For instance, multistability was found in phase oscillator networks with symmetric as well as asymmetric phase difference-dependent plasticity (a time continuous approximation of STDP; Seliger et al., 2002; Maistrenko et al., 2007) as well as in phase oscillator networks with STDP (Tass and Majtanik, 2006) and, subsequently, in different types of neuronal networks with STDP (Tass and Hauptmann, 2006; Popovych and Tass, 2012).

Based on a computational approach aiming at the development of stimulation techniques that specifically counteract abnormal neuronal synchrony by desynchronization (Tass, 1999), finally Coordinated Reset (CR) stimulation was designed (Tass, 2003a,b). CR stimulation means that phase resetting signals are delivered to different sub-populations within the abnormally synchronized neural network in a spatiotemporal manner (Tass, 2003a,b), such that the phase of each subpopulation is reset once within a stimulation ON-cycle. Computational studies showed that CR stimulation in a network with STDP (Gerstner et al., 1996; Markram et al., 1997; Bi and Poo, 1998; Feldman, 2000) not only causes a desynchronization of the abnormally strong synchronized neuronal activity but due to STDP also decreases the average synaptic weight. This anti-kindling, i.e., unlearning of abnormal synaptic connectivity and of abnormal neuronal synchronized activity by repetitive stimulation, is a long-lasting sustained effect (Tass and Majtanik, 2006; Hauptmann and Tass, 2007, 2009; Popovych and Tass, 2012). Abnormal synaptic connectivity in this context means abnormally strong synaptic connectivity that induces abnormal neuronal synchrony. The anti-kindling approach is not restricted to epilepsy. Rather it was designed to be tested in a number of disease conditions characterized by abnormally strong neuronal synchrony. In addition, computational studies showed that anti-kindling can robustly be obtained in networks with plastic excitatory and inhibitory synapses, no matter whether CR stimulation is delivered directly to the soma or via synapses (Popovych and Tass, 2012; Tass and Popovych, 2012). In line with these computational findings, long-lasting CR-induced anti-kindling was achieved invasively as well as non-invasively: in pre-clinical and clinical studies with rat hippocampal slices rendered epileptic by magnesium withdrawal (Tass et al., 2009), with parkinsonian non-human primates (Tass et al., 2012b), and with parkinsonian patients (Adamchic et al., 2014a), as well as in a proof of concept-study with tinnitus patients (Tass et al., 2012a; Adamchic et al., 2012a,b, 2014b; Silchenko et al., 2013).

Under certain circumstances, like in the case of side effects, such as speech and gait deterioration in Parkinson's patients receiving deep brain stimulation (Mahlknecht et al., 2015) or in tinnitus patients suffering from hyperacusis (Sheldrake et al., 2015), it might be desirable or even unavoidable to use weak stimulation intensities for the CR stimulation. For instance, to date the goal of clinical programming recipes for traditional, permanent high-frequency deep brain stimulation is to select stimulation sites and parameters such that target regions are maximally covered, and stimulation of adjacent regions is minimized (Saint-Cyr et al., 2000; Dujardin et al., 2001; Schroeder et al., 2003; Rodriguez-Oroz et al., 2005; Deuschl et al., 2006a; Hershey et al., 2008; van Nuenen et al., 2008; McIntyre et al., 2011; Mikos et al., 2011; Xu et al., 2011; Homer et al., 2012; Tommasi et al., 2012; Pinsker et al., 2013; Jahanshahi et al., 2015). Accordingly, to adapt the spatial extent of current spread according to anatomical borders of stimulation targets new electrode designs were suggested (Martens et al., 2011; van Dijk et al., 2015). However, there are side effects that are at least partly due to stimulation of the sensorimotor target region itself (Moreau et al., 2008; Jahanshahi et al., 2015); Accordingly, in addition to spatially shaping stimulation currents according to anatomical borders of target regions, we pursue an approach that tends to minimize side effects by using qualitatively different stimulation patterns which induce therapeutic effects that outlast cessation of stimulation and require weaker intensities compared to standard deep brain stimulation, e.g., a third of the standard stimulation amplitude (Tass et al., 2012a). In this study, we want to drive our approach forth with the final goal to enable clinically effective stimulation at further and substantially reduced stimulation intensities. CR stimulation essentially requires to stimulate neuronal subpopulations in a sufficiently separated manner. Hence, for a given, fixed spatial alignment of the stimulation sites, like in the case of deep brain stimulation, an appropriate stimulation amplitude has to be chosen in order to avoid a global, spatially non-specific stimulation and rather manipulate the different subpopulations separately (Tass et al., 2012b). The situation is different and more complex if stimulation sites can easily be modified as, e.g., in the case of acoustic CR stimulation for the treatment of tinnitus. In that case, not only stimulation intensity, but also stimulation sites have to be calibrated properly. For instance, based on the tonotopic organization of the central auditory system, tones are audiologically calibrated to the tinnitus pitch (Tass et al., 2012b), and a lot of efforts were put into improving reliable audiological calibration procedures (Hauptmann et al., 2016).

Computational studies have shown that CR stimulation is most effective for intermediate stimulation intensities when applied with rapidly varying sequences (RVS; Lysyansky et al., 2011; Popovych and Tass, 2012; Ebert et al., 2014; Zeitler and Tass, 2015) as well as with slowly varying sequences (SVS; Zeitler and Tass, 2015). In this study a *sequence* denotes the sequence of activating the spatially evenly distributed stimulation sites within the neuronal population once during the stimulation CR-on cycle. For the RVS CR stimulation the sequence changes continuously, whereas for the SVS-*n* CR stimulation the sequence is repeated *n*-times before another sequence is applied. For intermediate stimulation intensities the SVS CR stimulation, with its many repetitions of each sequence is more effective and robust in inducing an anti-kindling than the completely random RVS CR stimulation. However, for weak stimulation intensities the probability that a pronounced anti-kindling is achieved is higher for the RVS than for the SVS CR stimulation. So, replacing the commonly used RVS CR by the SVS CR is rather a disadvantage at weak stimulation intensities. However, these numerical results led us to the idea of sequentially combining the two CR protocols by delivering a two-stage CR stimulation in which after a preparatory stimulation at weaker intensities we switch over to a different CR protocol at intermediate intensities.

In this study we set out to improve the CR stimulation protocol for weak stimulation intensities, so that the same amount of anti-kindling can be induced as for intermediate stimulation intensities. We do this by studying systematically the anti-kindling impact of the two-stage CR stimulation, which we compare with the impact of the single-stage CR stimulation. The latter contains only one CR stimulation type. In addition, we also study the anti-kindling effect of stepwise increase of the stimulation intensity during the stimulation period while retaining the same CR protocol (CR stimulation with weak onset). For comparison and illustration, as control condition we use sham stimulation, i.e., we deliver no stimulation at all. Sham stimulation means that a medical device in controlled clinical studies or a stimulator in a pre-clinical study act as a placebo or stim off control, see e.g., McCracken and Kiss (2014). We use this (in the context of our computational study) simple control condition, since already comparatively uncomplex stimulation protocols such as periodic stimulation delivered through one stimulation side may elicit complex dynamics (Popovych and Tass, 2011).

To investigate the effects of a two-stage CR stimulation, we performed numerical simulations of Hodgkin-Huxley neurons in a network which incorporates excitatory as well as inhibitory spike timing-dependent plasticity (Section Materials and Methods). First, we demonstrate the extent of anti-kindling as a function of the stimulation intensity for the single-stage RVS as well as for the single-stage SVS CR stimulation (Section Short Single-Stage CR-Stimulation). Subsequently, we compare the effects of single-stage CR stimulation with weak onset and two-stage CR stimulation with weak onset (section Single- and Two-Stage CR Stimulation with Weak Onset). In Section Single- and Two-Stage CR Stimulation with Weak Onset we also attempt to disentangle the roles played by the individual CR approaches for very weak and weak stimulation intensities. We compare, the anti-kindling induced by short and long single-stage CR stimulation as well as by the two-stage CR-stimulation for different stimulation intensities (Section Two-Stage CR Stimulation with Constant Stimulation Intensity). Finally, we discuss possible clinical applications of the two-stage CR approach presented here.

## Materials and methods

In this section, we describe the equations used to model the neuronal and synaptic dynamics, the characteristics of the RVS and the SVS CR stimulation signals including the equations used to model the CR-stimulation, additional simulation details as well as the methods used for data analysis.

### Conductance-based hodgkin-huxley neuron model

The conductance-based Hodgkin-Huxley neuron model (Hodgkin and Huxley, 1952) used in this work is that of Hansel et al. (1993). For clarity we briefly recapitulate part of their model here. The differential equation of the membrane potential is given by:

(1) $$\begin{array}{rcl} C\frac{dV}{dt} & = & \;I-g_{Na}m^{3}h\left(V-V_{Na}\right)-g_{K}n^{4}\left(V-V_{K}\right)\\ -g_{l}\left(V-V_{l}\right)+I\left(t\right). \end{array}$$

The letters *C, V, t, g, I*, and *I*(*t*) denote membrane capacitance, membrane potential, time, maximum conductance for the ion or leak channel, the constant depolarizing current injected into the neuron which fixes the intrinsic firing rate of the neuron, and a time-varying current. The latter will be described in Equations (4), (10), and (11). The sodium and potassium reversal potentials are V_Na_ = 50 mV and V_K_ = −77 mV, and the leak reversal potential is V_l_ = −54.4 mV. The maximum conductances per unit area for the sodium, potassium, and leak currents are *g*_*Na*_ = 120 mS/cm^2^, *g*_*K*_ = 36 mS/cm^2^, and *g*_*l*_ = 0.3 mS/cm^2^. Other values used in this article are *C* = 1 F/cm^2^ and *I*∈[*I*_0_ − ε_*I*_, *I*_0_ + ε_*I*_] with *I*_0_ = 11.0 A/cm^2^ and ε_*I*_ = 0.45 A/cm^2^. See Hansel et al. (1993) or Popovych and Tass (2012) for the equations of the time-varying gate variables *m, h*, and *n*. In this study the initial conditions of all N neurons were randomly drawn from uniform distributions (*n*_*i*_, *m*_*i*_, *h*_*i*_, *s*_*i*_ ∈[0, 1];*V*_*i*_ ∈ [−65, 5]*mV*; *I*_*i*_ ∈ [*I*_0_ − σ_*I*_, *I*_0_ + σ_*I*_]). We define the “spike times” of the neuron as times at which *V* = 0.0 mV and *dV*∕*dt* < 0 mV/ms.

### Network

We use the network model described in Popovych and Tass (2012), which we briefly summarize here. The 1D neural network consists of *N* = 200 conductance-based Hodgkin-Huxley neurons and has periodic boundary conditions to minimize boundary effects. The neurons are initially all-to-all coupled with short-range strong excitatory and long-range weak inhibitory synaptic couplings according to a Mexican Hat (Wilson and Cowan, 1973; Dominguez et al., 2006; De la Rocha et al., 2008) like a weighting factor *M*_*ij*_ for the synaptic coupling from neuron *j* to *i*. The spatial profile of coupling is given by:

(2) $$\begin{array}{r} M_{ij}=\left(1-d_{ij}^{2}∕\sigma_{1}^{2}\right)exp\left(-d_{ij}^{2}∕\left(2\sigma_{2}^{2}\right)\right), \end{array}$$

where *d*_*ij*_ = *d*|*i*−*j*| denotes the distance between neurons *i* and *j*, and

(3) $$\begin{array}{r} d=d_{0}∕\left(N-1\right), \end{array}$$

the lattice distance between adjacent neurons within the ensemble. The length of the neuronal chain is given by *d*_0_ (*d*_0_ = 10), σ_1_ = 3.5, and σ_2_ = 2.0 as used in Popovych and Tass (2012).

Each neuron *i* receives post-synaptic currents (PSCs) from the other neurons in the network. The weighted ensemble average of all PSCs received by neuron *i* is (part of) the time-varying current *I(t)* (see Equation 1) and given by the coupling term *S*_*i*_ (Popovych and Tass, 2012)

(4) $$\begin{array}{r} S_{i}=N^{-1}\overset{N}{\underset{j=1}{\sum }}\left(V_{r,j}-V_{i}\right)c_{ij}|M_{ij}|s_{j}, \end{array}$$

where *N* is the number of neurons, *V*_*r, j*_ is the reversal potential of the synaptic coupling (20 mV for excitatory and - 40 mV for inhibitory synapses), *V*_*i*_ is the membrane potential of neuron *i*, and *c*_*ij*_ is the synaptic coupling strength from neuron *j* to neuron *i*. There are no self-connections within the network. In this study the initial synaptic weights c_ij_ between the neurons were drawn from a normal distribution ($c_{ij}\sim N\left(\mu =0.5\;\mu {\text{A}∕\text{cm}}^{2},\sigma =0.01\;\mu {\text{A}∕\text{cm}}^{2}\right)$)

The synaptic variable *s*_*j*_ is given by

(5) $$\begin{array}{r} \frac{ds_{j}}{dt}=\;\frac{0.5\left(1-s_{j}\right)}{1+exp\left[-\left(V_{j}+5\right)∕12\right]}-2s_{j}. \end{array}$$

### Spike timing-dependent plasticity

In general, synapses are dynamic and change their connection strengths depending on the precise timing of pre-and post-synaptic spikes (Markram et al., 1997; Bi and Poo, 1998). All synapses within the network are sensitive to the time difference (△*t*_*ij*_) between post- and pre-synaptic spike times *t*_*i*,_ respectively, *t*_*j*_. According to the spike timing-dependent plasticity (STDP) learning rule (Bi and Poo, 1998) the changes in connection strength is given by

(6) $$△c_{ij}=\{\begin{array}{cc} \beta_{1}e^{\frac{-△t_{ij}}{\gamma_{1}\tau }}, & △t_{ij}\geq 0\\ \beta_{2}\frac{△t_{ij}}{\tau }e^{\frac{△t_{ij}}{\gamma_{2}\tau }}, & △t_{ij}<0 \end{array}$$

(see Popovych and Tass, 2012) where △*t*_*ij*_ = *t*_*i*_ − *t*_*j*_ is the difference between post-synaptic spike time *t*_*i*_ and pre-synaptic spike time *t*_*j*_. Synaptic weights *c*_*ij*_ are updated in an event-based manner by adding δ·△*c*_*ij*_ for excitatory connections and −δ·△*c*_*ij*_ for inhibitory connections with learning rate δ>0 every time a neuron spikes. To avoid an unbounded strengthening or weakening, the synaptic weights are restricted to the interval *c*_*ij*_ ∈ [0, 1] mS/cm^2^. In this study we use values for the STDP parameters as in Popovych and Tass (2012): β_1_ = 1, β_2_ = 16, γ_1_ = 0.12, γ_2_ = 0.15, τ = 14 ms, and δ = 0.002.

### Coordinated reset

The CR stimulation was delivered to the network in a spatiotemporal manner via *N*_*s*_ equidistantly spaced stimulation sites (Tass, 2003a). These stimulation sites are activated one after the other, such that after one stimulation ON-cycle of duration *T*_*s*_ each stimulation site is stimulated exactly once. This spatiotemporal activation of stimulation sites is represented by the indicator functions ρ_*k*_(*t*) (*kϵ* {1, …, *N*}):

(7) $$\rho_{k}\left(t\right)=\{\begin{array}{cc} 1, & k^{th}\text{ stimulation site is active at }t\\ 0, & \text{otherwise} \end{array}.$$

These CR stimulation signals induce single brief excitatory PSCs, which evoke post-synaptic normalized conductances in the post-synaptic membrane. The conductances are represented by α-functions (Popovych and Tass, 2012 given by):

(8) $$\begin{array}{r} G_{stim}\left(t\right)=\frac{t-t_{k}}{\tau_{stim}}e^{-\left(t-t_{k}\right)∕\tau },\;t_{k}\leq t\leq t_{k+1}, \end{array}$$

where τ_*stim*_ = *T*_*s*_ ∕ (6*N*_*s*_) is the time-to-peak of G_stim_, and t_k_ is the onset of the k^th^ activation of the stimulation site. The spatial spread of the induced excitatory PSCs in the network is defined by a quadratic spatial decay profile (see Popovych and Tass (2012) for motivation):

(9) $$\begin{array}{r} D\left(i,x_{k}\right)=\frac{1}{1+d^{2}{\left(i-x_{k}\right)}^{2}∕\sigma_{d}^{2}}, \end{array}$$

were d denotes the lattice distance between two neighboring neurons as defined in Equation 3, *i* is the index of neuron *i*, *x*_*k*_ is the index of the neuron at stimulation site *k*, and σ_*d*_ = 0.08 *d*_0_ denotes the spatial decay rate of the stimulation current.

The current evoked in neuron *i* by the CR stimulation with stimulation intensity *K*, is defined like in Popovych and Tass (2012):

(10) $$\begin{array}{r} F_{i}=\left[V_{r}-V_{i}\left(t\right)\right]\cdot K\overset{N_{s}}{\underset{k=1}{\sum }}D\left(i,x_{k}\right)\rho_{k}\left(t\right)G_{stim}\left(t\right), \end{array}$$

where *V*_*r*_ = 20 mV denotes the excitatory reverse potential, V_i_ the membrane potential of neuron *I*, and ρ, *G, D* are given by Equations (7), (8), and (9). This implies that during the period with CR stimulation (briefly CR-on period) the total time-dependent current *I(t)* of neuron *i* in Equation 1 is the sum of the coupling term and the stimulation current

(11) $$\begin{array}{r} I_{i}\left(t\right)=S_{i}\left(t\right)+F_{i}\left(t\right). \end{array}$$

**Figure 1:**
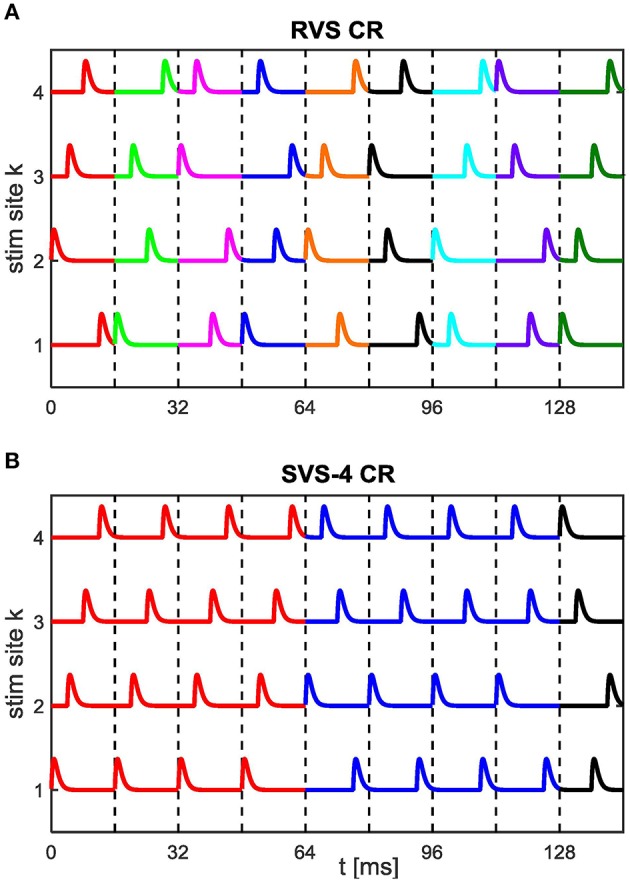
**Spatiotemporal stimulation signals of CR stimulation**. An example sequence order for the **(A)** RVS CR stimulation with a new randomly drawn sequence for each stimulation ON-cycle and for the **(B)** SVS CR stimulation with every randomly drawn sequence repeated during four consecutive stimulation ON-cycles (SVS-4) before the next sequence is drawn. Vertical dashed lines separate stimulation ON-cycles. Newly drawn sequences are represented by a different color. In this study the CR-on periods exclusively contain stimulation ON-cycles: no stimulation OFF-cycles. For the remainder of this study each sequence is repeated consecutively 100 times in the SVS CR approach.

During the period after the CR stimulation is switched off (briefly CR-off period) *F(t)* = 0. In this paper we use the RVS CR (Tass and Majtanik, 2006; Tass and Hauptmann, 2006; Popovych and Tass, 2012; Tass and Popovych, 2012), and the SVS CR (Zeitler and Tass, 2015). A stimulation site sequence, briefly *sequence*, is the spatiotemporal sequence by which all stimulation sites are stimulated exactly once. In the RVS CR approach, the sequences are drawn randomly (see Figure 1A). In the SVS-*n* CR approach a randomly drawn sequence is consecutively repeated *n*-times before the next sequence is randomly drawn (see Figure 1B for *n* = 4). In this paper we use *n* = 100. Since *n* is constant in this study we will write SVS instead of SVS-100 for the remainder of this study. The stimulation sites used in this study are located at neuron indices 25, 75, 125, and 175.

### Simulation details

After an initial equilibration period of 2 s, STDP was included for the rest of the simulation. During the first 60 s with STDP the network rewires its connections. At the end of this STDP-only period the network activity is highly synchronized and at *t* = 0 ms the CR simulation is applied for the duration of the total CR-on period, *T*_*CR*−*on*_. In this study this CR-on period consists only of stimulation ON-cycles, each lasting *T*_*s*_ = 16 ms. After *T*_*CR*−*on*_ the single-stage CR-stimulation is stopped permanently and the CR-off period starts. The short single-stage CR-stimulation lasts for *T*_*CR*−*on*_ = 64 s, the other single-stage CR-stimulation are twice as long (*T*_*CR*−*on*_ = 128 s). The CR-off period lasts as long as the CR-on period.

For comparison a sham stimulation is used as control stimulation: during sham stimulation no stimulus is delivered, so that this control condition is just a continuation of the STDP-only period since the stimulation intensity, *K* equals zero.

For the two-stage CR-stimulation the first CR-on period starts at *t* = 0 s and lasts for 64 s. At *t* = 64 s the first CR stimulation is switched off and the second CR-stimulation is applied. At *t* = 128 s the second CR-stimulation is then switched off, and the 128 s lasting CR-off period starts. The stimulation intensity of the CR-stimulation during the *i*-th CR-on period is denoted as *K*_*i*_, the CR-approach during the *i*-th CR-on period of the two-stage CR as RVS_*i*_ if RVS CR was applied and SVS_*i*_ in case of SVS CR (with ∈{1, 2}). For the single-stage CR it is also possible to have two CR-on periods with different stimulation intensities *K*_*1*_ and *K*_*2*_. However, by definition only one CR approach is used in the single-stage CR and therefore, the CR-approach is denoted without index *i*, e.g., simply as RVS or SVS CR.

The initial network conditions as well as the sequence order have an influence on the anti-kindling (Zeitler and Tass, 2015). Therefore, eleven simulations are executed for the same CR-protocol and stimulation intensity, *K*, but for different combinations of initial network conditions and sequence orders, hereafter referred to as “*samples*.” The obtained distributions for *C*_*av*_ and *R*_*av*_ are represented in boxplots (Tukey, 1977).

All simulations were done in Matlab R2007a. The differential equations were solved by the built-in function ODE45 with a relative tolerance of 10^−5^.

The present study is part of a series of studies addressing different topics and issues investigated with the same numerical model. As soon as the related papers will be in press, the Matlab Code will be uploaded to ModelDB (https://senselab.med.yale.edu/ModelDB/).

### Data analysis

In plastic neural networks CR stimulation can lead to anti-kindling, i.e., an unlearning of abnormal synaptic connectivity and abnormal synchronization. In this study the synaptic weights change constantly due to STDP and the different intrinsic firing rates of the neurons. Therefore, the dynamics of the synaptic connectivity is monitored on a population level by the synaptic weight averaged over the population:

(12) $$\begin{array}{r} C_{av}\left(t\right)=N^{-2}\underset{i,j}{\sum }sgn\left(M_{ij}\right)c_{ij}\left(t\right), \end{array}$$

where *M*_*ij*_ as defined in Equation 2, *sgn* denotes the sign-function and *c*_*ij*_ is the synaptic coupling strength from neuron *j* to neuron *i*.

The degree of synchronization of the neuronal activity is influenced by synaptic weights and can be measured by the order parameter *R* (Haken, 1983; Kuramoto, 1984)

(13) $$\begin{array}{r} R\left(t\right)\text{exp}\left[i\Phi \left(t\right)\right]=N^{-1}\overset{N}{\underset{j=1}{\sum }}\text{exp}\left[i\varphi_{j}\left(t\right)\right], \end{array}$$

where φ_*j*_(*t*) = 2π(*t*−*t*_*j, m*_)∕(*t*_*j, m*+1_ − *t*_*j, m*_) for *t*_*j, m*_ ≤ *t*<*t*_*j, m*+1_ is a linear approximation of the phase of neuron *j* between its *m*^*th*^ and (*m*+1)^*th*^ spikes at spike times *t*_*j, m*_ and *t*_*j, m*+1_. A phase in case of a rhythmically active neuron can be approximated along the lines of the phase oscillator concept (Kuramoto, 1984; Tass, 1999) or simply by a piece-wise linear function of time (Rosenblum et al., 2001). Φ(*t*) is the circular mean phase of the entire group of *N* neurons. An *R*-value of zero indicates a complete desynchronization and a value of one indicates perfect in-phase synchronization. For our data analysis the order parameter is averaged over the last 1.6 s of the CR-off period and is called the average order parameter *R*_*av*_.

Statistical significances of differences between the obtained distributions for *C*_*av*_ and *R*_*av*_ (represented in boxplots) are determined by the one-sided Mann-Whitney-U- test with *p* < 0.05.

To investigate the mechanisms of RVS and SVS CR stimulation we study the stimulus-locked dynamics on a mesoscopic scale by considering subgroups of neurons as given by their proximity to the four different stimulation sites. The latter are located at neuron indices 25, 75, 125, and 175. Accordingly, we divide the whole population into 4 subgroups comprising *N*/*N*_*s*_ = 50 neurons each, where the number of stimulation sites is given by *N*_*s*_ = 4. The mean phase of subgroup *sg*, Φ_*sg*_(*t*) is determined by

(14) $$\begin{array}{l} R_{sg}\left(t\right)\text{exp}\left[i\Phi_{sg}\left(t\right)\right]=\;N_{sg}^{-1}\sum_{j=1}^{N_{sg}}\text{exp}\left[i\varphi_{j}\left(t\right)\right], \end{array}$$

where the summation runs only over the neurons within subgroup *sg*, and *sg* = 1, …, 4. We perform a cross-trial analysis of the stimulus responses of each subgroup separately. In particular, we focus on the stimulus-locked phase dynamics of the subgroups, to reveal e.g., phase resetting or entrainment processes that are tightly time locked to the repetitively administered stimuli. To study the phase dynamics of a subgroup in an ensemble of stimulus-locked responses for each time Δt in a time window *W* = [–32 ms, +32 ms] attached to each stimulus onset τ_k_ we determine the *cross-trial distributions* of the phases of the different subgroups

(15) $$\begin{array}{l} {\left\{\Phi_{sg}\left(\tau_{k}^{sg}+\Delta t\right)\left(\text{mod }2\pi \right)\right\}}_{k=1,\ldots ,L} \end{array}$$

where $\tau_{k}^{sg}$ denotes the onset of the *k*-th stimulus delivered to subgroup *sg*, and L is the total number of stimuli delivered to the subgroup *sg* (Tass, 2003d). These cross-trial distributions will be displayed in a color plot as a function of △*t*∈*W*. In our data we only found phase distributions that were either close to uniform or had (only) one pronounced peak. Accordingly, to quantify the amount of stimulus-locking of the phase dynamics, we use the resetting index *E*_*sg*_(△ *t*) of subgroup *sg* defined by

(16) $$E_{sg}\left(△t\right)=\;\left|L^{-1}\sum_{k=1}^{L}\text{exp}\left[i\Phi_{sg}\left(\tau_{k}^{sg}+\Delta t\right)\right]\right|,$$

where *L* is the number of administered stimuli (Tass, 2003d; see also Tallon-Baudry et al., 1996). If the phase dynamics is not stimulus-locked at time △t, the corresponding phase distribution is uniform. Otherwise, one observes a distribution that significantly differs from a uniform distribution and has, e.g., one dominant peak. For instance, in case of a stimulus-induced phase reset the phase distribution will turn from uniform in the pre-stim period to unimodal after stimulus onset (Tallon-Baudry et al., 1996; Tass, 2003d). In contrast, a permanent stimulus-locking of the phase dynamics in terms of a phase entrainment can show up as unimodal distribution throughout the whole time window W.

As a motivation for the subsequent analysis, let us consider possible differences of the mechanisms and effects of RVS CR and SVS CR:

*SVS CR (during an epoch with a constant sequence):* Each subgroup is stimulated periodically. Periodic stimulation of an isolated (i.e., not synaptically interacting) subgroup may cause an entrainment that may lead to an increase of the synchronization, provided stimulation is strong enough. SVS CR (with constant sequence) delivered to mutually isolated subgroups may, thus, cause a phase-shifted entrainment of the different subgroups at high enough stimulation intensities. The mutual synaptic interaction of the different subgroups may cause a perturbation of the respective entrainment processes. Note, the effect of periodic stimulation on an isolated subgroup may be complex, as shown e.g., in the context of periodic stimulation of neurons interacting through excitatory synapses (Popovych and Tass, 2011): Depending on the mismatch of intrinsic frequencies and stimulation frequency periodic stimulation may cause a desynchronization—not just an entrainment. Accordingly, it is not justified to simplify the mechanism of SVS CR as being a combination of exclusively synchronizing direct effects of stimulation and desynchronizing indirect, synaptically mediated effects.

*RVS CR:* The direct stimulation effect on each subgroup is fundamentally different compared to SVS CR, since it is not a periodic stimulation.

By design, SVS CR and RVS CR stimulation affect subgroups differently: Accordingly, we study synchronization processes not only on the macroscopic level of the entire neuronal population, but also on the mesoscopic level of the different subgroups. To assess the amount of synchronization of the *j*-th subgroup we introduce

$$\begin{array}{lll} {\bar{R}}_{j}^{pre} & = & <R_{j}{>}_{last\;10\;s\;before\;CR-on}\\ {\bar{R}}_{j}^{on} & = & <R_{j}{>}_{last\;10\;s\;CR-on}\\ {\bar{R}}_{j}^{off} & = & <R_{j}{>}_{first\;10\;s\;CR-off} \end{array}$$

where *R*_*j*_ denotes the synchronization order parameter of the *j*-th subgroup as determined by Equation 14. The acute CR effect on the *j*-th subgroup is given by

(17) $$\begin{array}{l} 1-\frac{{\bar{R}}_{j}^{on}}{{\bar{R}}_{j}^{pre}} \end{array}$$

whereas the acute after-effect on the *j*-th subgroup reads

(18) $$\begin{array}{l} 1-\frac{{\bar{R}}_{j}^{off}}{{\bar{R}}_{j}^{pre}}, \end{array}$$

where an outcome of zero means no acute CR effect (after-effect, respectively), a positive outcome indicates a desynchronizing and a negative value a synchronizing effect on the *j*-th subgroup due to CR stimulation.

Since CR stimulation induces phase shifts between the different subgroups, we study synchronization processes between the different subgroups by calculating the phase difference between the *j*-th subgroup and the *k*-th subgroup defined by

(19) $$\begin{array}{l} \vartheta_{jk}\left(t\right)=\;\Phi_{j}\left(t\right)-\Phi_{k}\left(t\right), \end{array}$$

with Φ_*sg*_(*t*) the mean phase of subgroup *sg* as determined by Eq. 14. We will determine the distribution of ϑ_*jk*_(*t*) during the last 10 s with CR-on as well as during the first 10 s with CR-off and show this distribution by an angular distribution plot with bins of π∕18 rad.

To detect whether a distribution of ϑ_*jk*_ has several peaks we introduce the indices

(20) $$\lambda_{jk}^{\left(\nu \right)}=\left|\frac{1}{P}\sum_{l=1}^{P}\text{exp}\left[i\nu \vartheta_{jk}\left(t_{l}\right)\right]\right|,$$

where *P* is the number of sampling points in the 10 s window (*P* = 10,000) and ν is the order of λ (Batschelet, 1981; Kuramoto, 1984; Tass, 1999, 2003d). Perfect phase synchronization between the *j*-th and *k*-th subgroup with ϑ_*jk*_(*t*) = *const* is associated with $\lambda_{jk}^{\left(\nu \right)}=1$ for ν = 1, 2, 3, … . Conversely, a perfect uniform distribution of ϑ_*jk*_ is related to $\lambda_{jk}^{\left(\nu \right)}=0$ for ν = 1, 2, 3, … . A bimodal distribution of ϑ_*jk*_ with anti-phase peaks can be detected with $\lambda_{jk}^{\left(2\right)}-\lambda_{jk}^{\left(1\right)}$ (Tass, 2003d). Given the numerical results obtained in the present study (see Results), we seek to detect distributions with three rectangularly aligned peaks located at π∕2, π and3π∕2. To this end, we introduce the index

(21) $$\begin{array}{l} \alpha_{jk}={\left[\lambda_{jk}^{\left(4\right)}-\lambda_{jk}^{\left(1\right)}\right]}_{+}, \end{array}$$

where [*z*]_+_, stands for the half–wave rectification operation: [*z*]_+_ = *z* if *z*>0 and [*z*]_+_ = 0 else.

To motivate the choice of α_*jk*_ and study its characteristics, let us consider two different families of synthetic phase difference distributions with (i) four equidistantly aligned peaks at 0, π∕2, π and 3π∕2 and (ii) three rectangularly aligned peaks located at π∕2, π and 3π∕2.

*(i) 4 equidistantly aligned peaks:* The distribution of the phase differences is given by ${\left\{\psi_{l}\left(m\right)\right\}}_{l=1}^{4Z}$, where *m* = 0, …, *Z* serves as parameter. The phase differences read ψ_*l*_(*m*) = ϱ_*l*_(*m*)+ξ_*l*_, with random variable ξ_*l*_ uniformly distributed and fulfilling −ε ≤ ξ_*l*_ ≤ ε, where the parameter ε determines how narrow the distribution is, and *l* = 0, …, 4*Z*. The symmetrical 4-peak distribution is created by

(22) $$\begin{array}{l} \varrho_{l}\left(m\right)=\left({\left[l\right]}_{\text{mod}4}-1\right)\frac{\pi }{2}\mu_{l}\left(m\right) \end{array}$$

where

(23) $$\mu_{l}\left(m\right)=\{\begin{array}{ll} 0\;: & l>4m\\ 1\;: & \text{else} \end{array}$$

determines which index groups of four actually contribute to a 4 peak distribution (in the case of μ_*l*_(*m*) = 1) or to a 1 peak distribution instead (in the case of μ_*l*_(*m*) = 0). The limiting cases are a perfect 1-peak (Dirac-type) distribution for *m* = 0 and ε = 0 and a perfectly symmetrical distribution with 4 rectangularly arranged peaks for *m* = *Z* and ε = 0. For each given m we determine the parameters

(24) $$\lambda^{\left(\nu \right)}(m)=\left|\frac{1}{4Z}\sum_{l=1}^{4Z}\text{exp}\left[i\nu \psi_{l}\left(m\right)\right]\right|$$

for ν = 1 and ν = 4 and calculate

(25) $$\begin{array}{l} \alpha \left(m\right)={\left[\lambda^{\left(4\right)}\left(m\right)-\lambda^{\left(1\right)}\left(m\right)\right]}_{+} \end{array}$$

**Figure 2:**
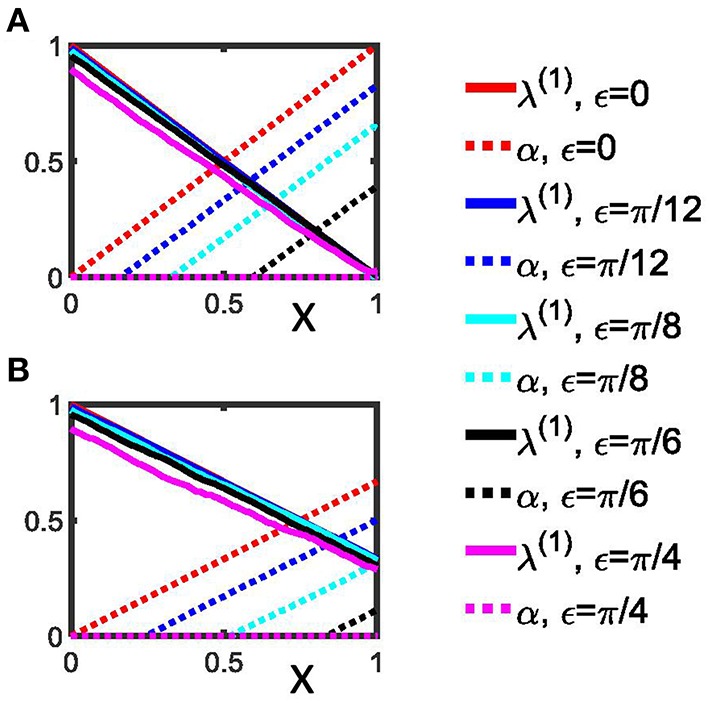
**Detection of rectangulary aligned peaks in a synthetic phase difference distribution. (A)** The first order indices λ^(1)^ (Equation 24) as well as the indices α (Equation 25) are shown as a function of x for the 4-peak phase difference distribution with different narrowness, ε, of the peaks. **(B)** Indices as in **(A)**, but determined by Equations 28 and 29 for the 3-peak phase difference distributions. Two extreme cases are shown: (i) perfect Dirac-type peaks (ε = 0); (ii) a homogenous distribution without any pronounced peaks (ε = π∕4). We used *Z* = 100 for both panels. See section Data analysis for more details.

For illustration we plot λ^(1)^(*m*) vs. *x* = *m*∕*Z* and α(*m*) vs. *x* = *m*∕*Z* (Figure 2A).

(ii) *Asymmetric 3-peak distribution:* For comparison, we consider the distribution given by ${\left\{\psi_{l}\left(m\right)\right\}}_{l=1}^{3Z}$, with parameter *m* = 0, …, *Z*. The phase differences are given by ψ_*l*_(*m*) = ϱ_*l*_(*m*)+ξ_*l*_, with *l* = 0, …, 3*Z*. The uniformly distributed random variable ξ_*l*_ is defined as above: −ε ≤ ξ_*l*_ ≤ ε. The rectangularly aligned 3 peaks of the distribution are generated by

(26) $$\begin{array}{l} \varrho_{l}\left(m\right)=\left({\left[l\right]}_{\text{mod }3}-1\right)\frac{\pi }{2}\mu_{l}\left(m\right) \end{array}$$

where

(27) $$\mu_{l}\left(m\right)=\{\begin{array}{ll} 0\;: & l>3m\\ 1\;: & \text{else} \end{array}$$

determines which index groups of three actually contribute to a 3-peak distribution [in the case of μ_*l*_(*m*) = 1] or to a 1 peak distribution instead [in the case of μ_*l*_(*m*) = 0]. Analogously to (i), the limiting cases are given by a perfect 1-peak (Dirac-type) distribution for *m* = 0 and ε = 0 and an asymmetric distribution with 3 peaks at π∕2, π, and 3π∕2 for *m* = *Z* and ε = 0. For each given m we determine the parameters

(28) $$\lambda^{\left(\nu \right)}(m)=\left|\frac{1}{3Z}\sum_{l=1}^{3Z}\text{exp}\left[i\nu \psi_{l}\left(m\right)\right]\right|$$

for ν = 1 and ν = 4. With this we obtain

(29) $$\begin{array}{l} \alpha \left(m\right)={\left[\lambda^{\left(4\right)}\left(m\right)-\lambda^{\left(1\right)}\left(m\right)\right]}_{+} \end{array}$$

For comparison, we plot λ^(1)^(*m*) vs. *x* = *m*∕*Z* and α(*m*) vs. $x=\frac{m}{Z}\;$(Figure 2B).

From Equations (24) and (28) it follows that in the case of 4 equidistantly aligned peaks as well as in the case of an asymmetric 3-peak distribution for ε = 0 the maximum of λ^(1)^ is attained for *x* = 0 and reads λ^(1)^ = 1. For ε = 0 the minimum of λ^(1)^ is reached at *x* = 1 and reads 0 in case of the symmetric 4-peak distribution and 1∕3 for the asymmetric 3-peak distribution. Hence, from Equations (25) and (29) we read off the maximum of α is reached for *x* = 1 for both distributions, but differs in size: α = 1 for the symmetric 4-peak distribution, but α = 2∕3 for the asymmetric 3-peak distribution (see Figure 2). Conversely, the minimum of α is 0 and attained for *x* = 0. Figure 2 illustrates how λ^(1)^ and α vary with increasing ε. A limiting case is ε = π∕4, which renders both distributions uniform, so that α = 0 for 0 ≤ *x* ≤ 1.

## Results

### Short single-stage CR-stimulation

**Figure 3:**
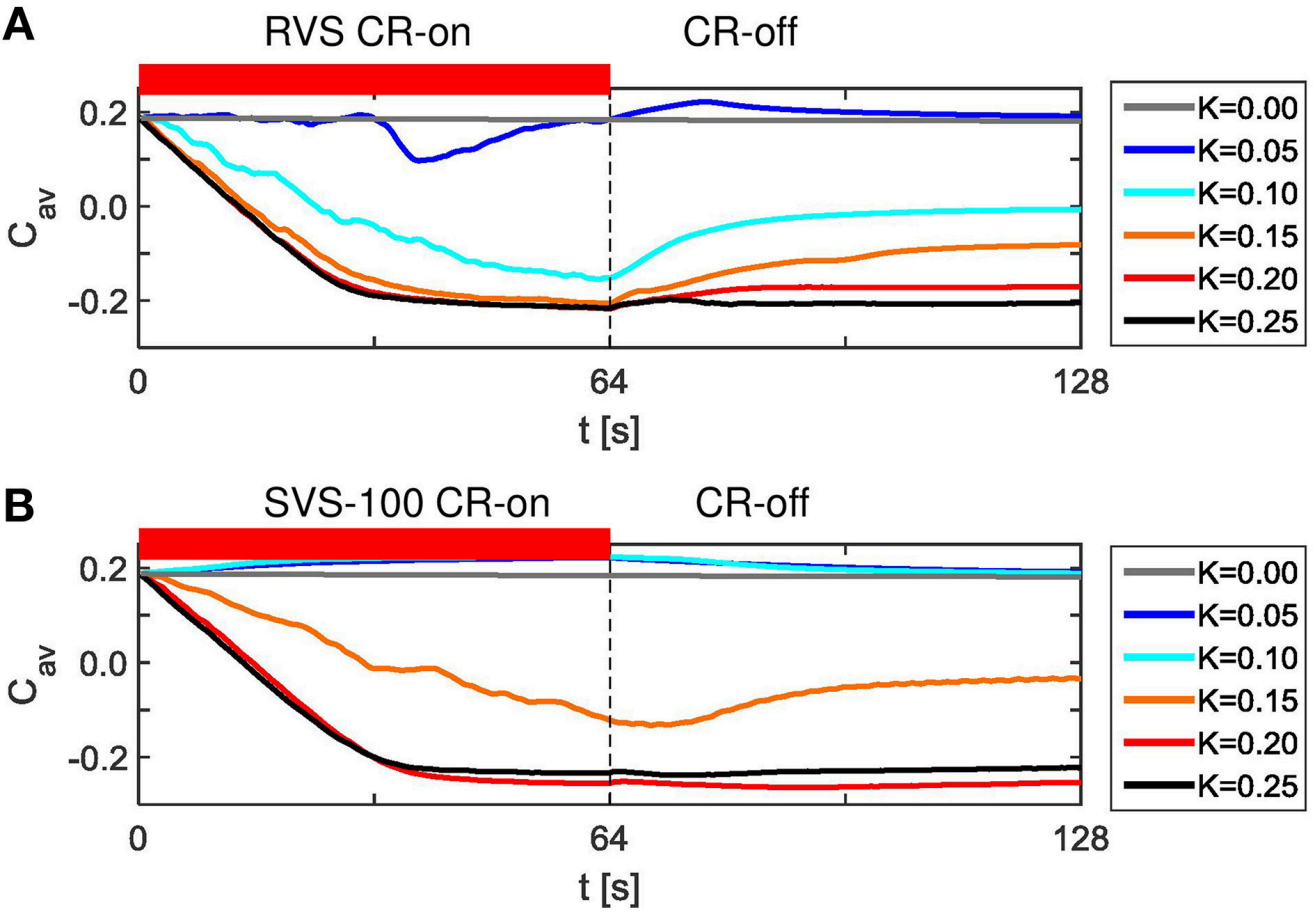
**Dynamics of the average synaptic weight, *C*_*av*_, for different stimulation intensities, *K*, for the short RVS and short SVS CR-stimulations. (A)** *C*_*av*_ as a function of time for the RVS CR with different stimulation intensities, *K*. **(B)** *C*_*av*_ dynamics for the SVS CR stimulation with different *K*. The red horizontal bar represents the CR-on period, which start at *t* = 0 s and is switched off at *t* = 64 s (dashed vertical line). No stimulation signals are delivered during the subsequent 64 s lasting CR-off period and *C*_*av*_ evolves spontaneously. The initial network is identical for all simulations. The sequence order of the RVS (SVS, respectively) CR stimulation is identical for all *K*-values.

Without stimulation (*K* = 0.0) the average synaptic weight, *C*_*av*_, does not change, but it decreases by the RVS CR stimulation during the CR-on period (Figure 3A). After switching off the CR stimulation the network evolves spontaneously. During this CR-off period the average synaptic weight remains more or less the same for certain stimulation intensities, for others it increases. Since the same sequence order is applied to the same initial network, the differences observed are only caused by the different stimulation intensities, *K*.

SVS CR with 100 consecutive repetitions also decreases the average synaptic weight, *C*_*av*_, for the stronger stimulation intensities, but for weak stimulation intensities it increases the average synaptic weight, *C*_*av*_, during the CR-on period (Figure 3B). For *K* = 0.05 and *K* = 0.10 the *C*_*av*_ dynamics are even almost identical and still different compared to the case without stimulation (*K* = 0.0). Again, the differences in Figure 3B are due to the different values of *K*, since one SVS sequence order is applied to the same initial network at different stimulation intensities. Since the initial network used for Figures 3A,B is identical, these figures suggest that for this particular initial network RVS CR is more effectively decreasing *C*_*av*_ for weak stimulation intensities, whereas SVS CR is more effective at stronger stimulation intensities.

**Figure 4:**
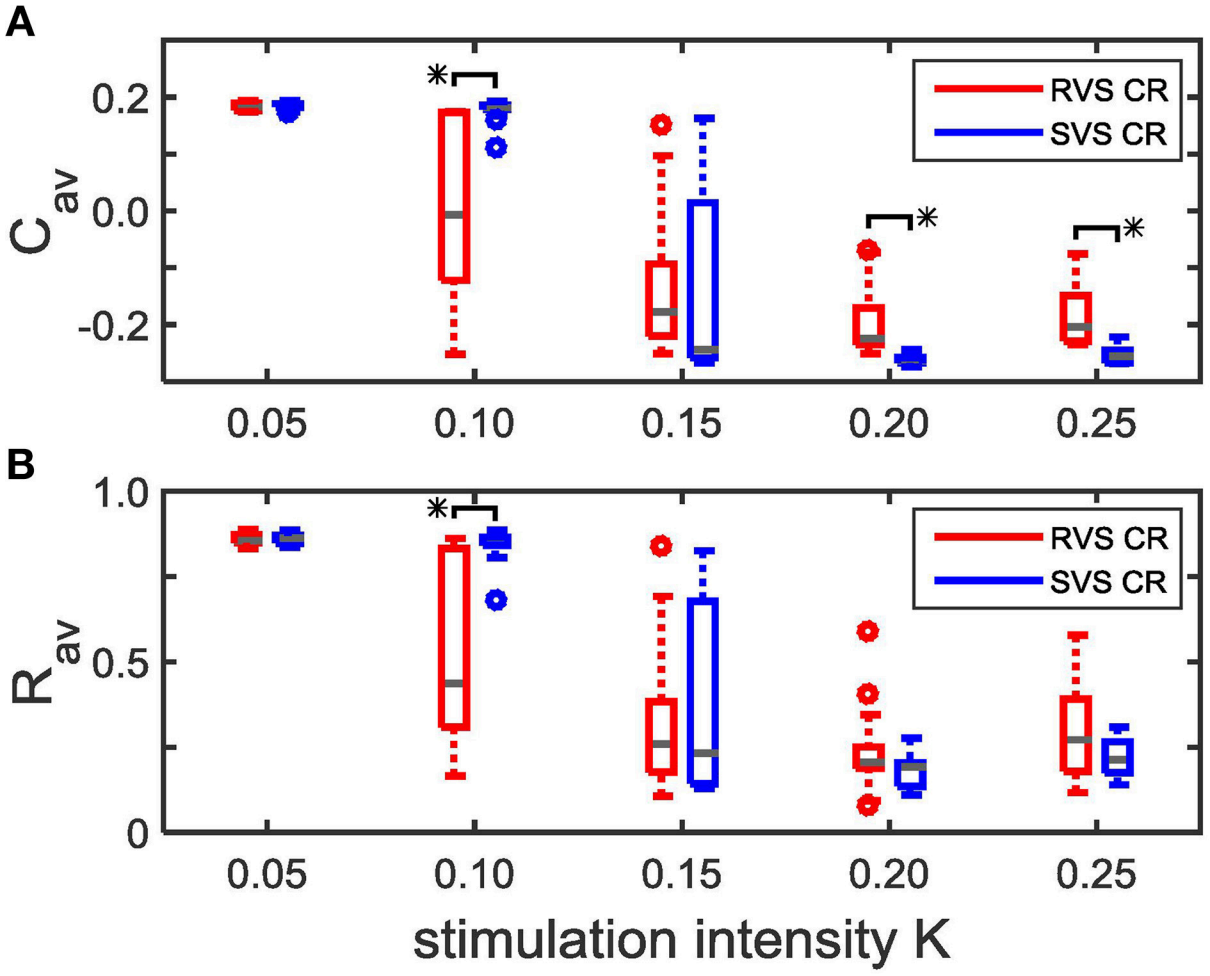
**Comparison of the average synaptic weight, *C*_*av*_, and the average order parameter, *R*_*av*_, at the end of the CR-off period (*t* = 128 s) for the short RVS and the short SVS CR stimulation. (A)** Boxplot of *C*_*av*_(*t* = 128 s) for different stimulation intensities, *K*, for the RVS (red) and SVS CR stimulation. **(B)** As **(A)** for the order parameter averaged over the last 1.6 s of the CR-off period, *R*_*av*_. For each condition (*K* value and CR approach) eleven samples are used. The distribution of the obtained *C*_*av*_ and *R*_*av*_ values are represented by a boxplot. The gray horizontal line within the box represents the median, the box itself the middle 50% and the whiskers below (above) the box the first (last, respectively) 25%. Outliers are defined as 1.5 times the length of the box below or above the box and represented by open circles. An asterisk indicates a statistically significant lower *C*_*av*_- or *R*_*av*_-value (one-sided Mann-Whitney test, *p* < 0.05).

Repeating the simulations for eleven samples (different combinations of initial networks and sequence orders) confirms that for *K* = 0.10 the RVS CR stimulation induces smaller *C*_*av*_ values than the SVS CR stimulation, and that for *K*≥0.20 the SVS CR induces smaller *C*_*av*_ values than the RVS CR stimulation. Both reductions of *C*_*av*_ are statistically significant (one-sided Mann-Whitney test, *p* < 0.05; Figure 4A). The average order parameter, *R*_*av*_, is smaller for the RVS than for the SVS CR for *K* = 0.10, and again this difference is statistically significant (one-sided Mann-Whitney test, *p* < 0.05; Figure 4B). Combining Figures 4A,B shows that for a stimulation intensity of *K* = 0.10 only the RVS CR approach can decrease the average synaptic weight and reduce the degree of synchrony of the neuronal activity. For stimulation intensities *K* ≥ 0.20 the SVS approach is superior with respect to a reduction of the mean synaptic connectivity. However, this does not induce a significantly stronger reduction of the mean synaptic connectivity.

**Figure 5:**
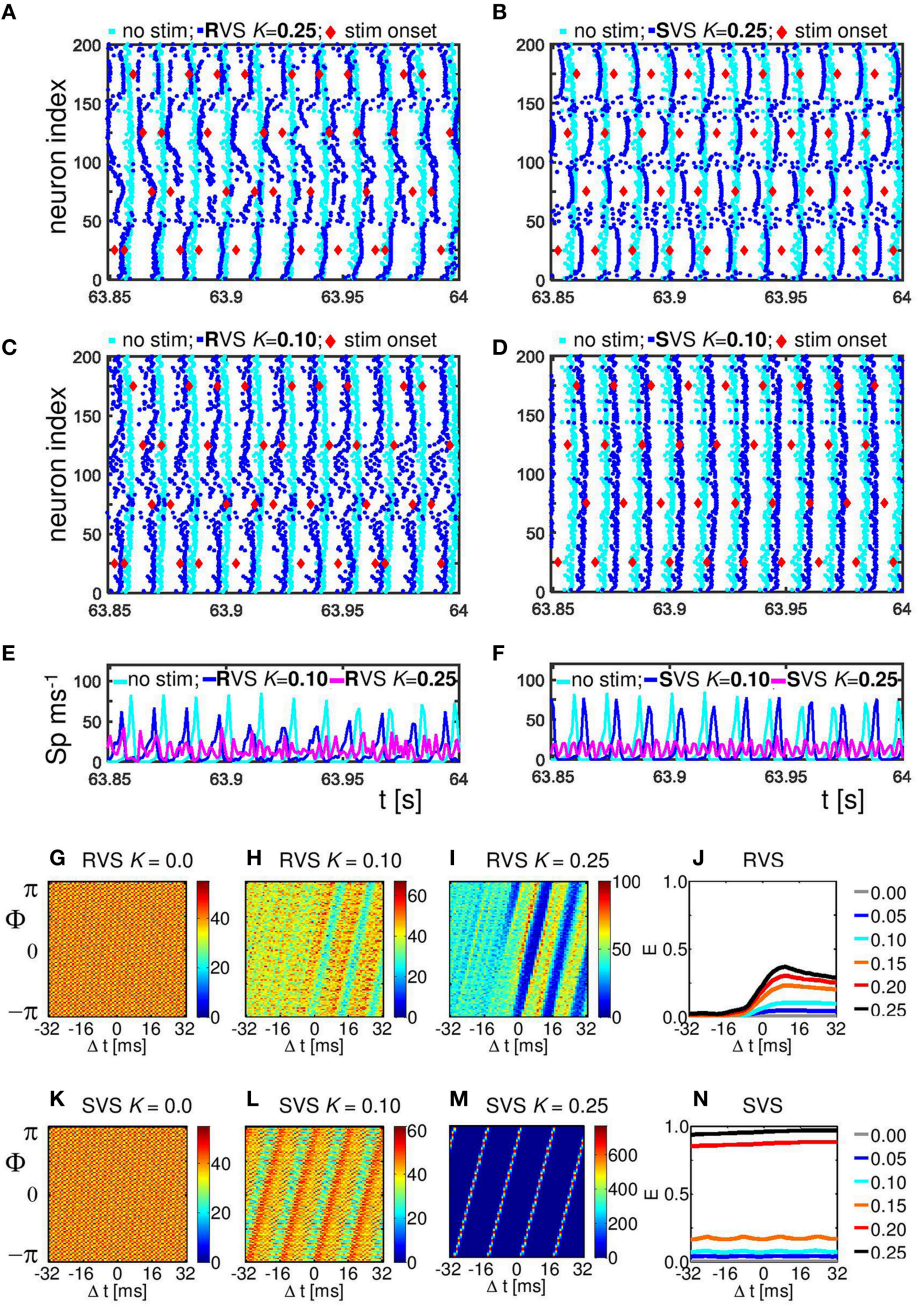
**Effect of RVS and SVS CR stimulation on the spike times at the end of the 64 s lasting CR-on period.(A)** Raster plot of the last 150 ms of the CR-on period with the RVS CR applied with stimulation intensity *K* = 0.25 (blue dots) and for the control stimulation with *K* = 0.00 (cyan dots). Each blue (cyan) dot represents the spike time of that particular neuron under the condition *K* = 0.25 (*K* = 0.00, respectively). **(B)** as **(A)** for SVS CR. **(C)** As **(A)** for *K* = 0.10. **(D)** as **(B)** for *K* = 0.10. The stimulation onset of the stimulation sites are represented by red diamonds in **(A–D)**. **(E)** Spike counts for the last 150 ms of the RVS CR-on period show how many neurons fire within each time interval of 1 ms. **(F)** as **(E)** for the SVS CR stimulation. **(G–I)** Distributions of the mean phase of neurons 51–100, Φ_sg = 2_(Δt), averaged across the 4000 activation times of subgroup 2 within the RVS CR-on period for different applied stimulation intensities. **(J)** Resetting index *E*(Δ*t*) of subgroup 2 determined for the RVS CR-on period for different stimulation intensities. **(K–M)** as **(G–I)** for the SVS CR stimulation. **(N)** as **(J)** for the SVS CR stimulation. For all panels are the same initial network conditions and sequence orders as used in Figure 3.

To study the mechanism by which CR stimulation desynchronizes the neuronal activity we compare the spike times of the individual neurons at the end of the CR-on period for a stimulation intensity *K* = 0.25, for which RVS as well as SVS CR stimulation work well, with our control stimulation (*K* = 0.00). The control stimulation (*K* = 0.00) “applied” to the initially synchronized network does not change the form of the spike volleys of the neurons (see Figures 5A–D). This is to demonstrate that the model does not change its dynamic mode spontaneously. By applying CR stimulation at an intensity *K* > 0 the network is stimulated in a spatiotemporal way: stimulation at different positions at different times as shown by the red diamonds in Figure 5. Stimulation (with *K* > 0) induces post-synaptic currents in the neurons. The amplitude of this induced current depends not only on the stimulation intensity, but due to the spatial decay profile (see Equation 9) also on the distance between the neuron and the stimulation site. Therefore, the spiking behavior of neurons near a stimulation site is more strongly affected as opposed to distant neurons. For our control stimulation the spike times of the neurons show some variations. The neurons are not in perfect synchrony, but display a certain time jitter. Due to the CR stimulation the spiking jitter increases, in particular, in neurons with conflicting impact from neighboring stimulation sites (see Figure 5A for RVS and Figure 5B for SVS CR stimulation). Since the inter-spike-intervals of strongly synchronized neurons are (almost) identical, this jitter can only be reduced if the neurons are all reset to a certain internal phase. To which state a neuron is reset depends on the timing of the stimulation in relation to the internal cycle of the neuron's dynamics. An excitatory stimulation of a Hodgkin-Huxley model neuron can advance or delay a spike: an excitatory stimulation received in the first part of the internal cycle will delay the next spike, in the last part of the internal cycle it will advance the spike time. This implies that a spike volley with some jitter can be split in two groups, usually these two groups will merge under the influence of further stimulation (see e.g., the neurons near stimulation site 175 in Figure 5A). So, the main reason why CR desynchronizes a neuronal population is because stimulation delivered at different sites within the network at different times divides the population into several phase-shifted synchronized subpopulations (located close to the stimulation sites) and groups of desynchronized neurons (located in between stimulation sites; see Figure 5A for the RVS CR stimulation and Figure 5B for the SVS CR stimulation).

By decreasing the stimulation intensity the induced synchronized subpopulations which are in general out-of-phase with each other, become smaller and will disappear first for the SVS and for weaker stimulation intensities also for the RVS CR stimulation. For *K* = 0.10 only the RVS CR is still able to divide the whole population into some desynchronized and some synchronized groups (Figure 5C). For the SVS CR stimulation a stimulation intensity of *K* = 0.10 is too weak to divide the whole population into distinct, phase-shifted groups (Figure 5D).

The spike count plots in Figures 5E,F show the number of spikes that are fired by the network within each time bin of size 1 ms. For the control stimulation there are high peaks, which can be reduced by the RVS CR stimulation applied at *K* = 0.10 (Figure 5E), but the SVS CR stimulation applied at the same intensity does not significantly reduce the overall synchronization (Figure 5F). However, for intermediate stimulation intensities like *K* = 0.25, both CR approaches desynchronize the neuronal network activity (Figures 5E,F)

The analysis with the cross-trial phase distributions, from Equation (15), reveals a qualitative difference of the effects induced by RVS as opposed to SVS CR stimulation: for sufficient stimulation strength the non-periodically delivered RVS stimuli cause phase resets, whereas the periodically delivered SVS stimuli induce an entrainment. The cross-trial phase distribution in a window from −32 to +32 ms relative to the stimulation onset of the site belonging to the neuronal subgroup comprising neurons 51–100 shows that for the sham stimulation (K = 0.0) the phase distribution is flat throughout the entire time window (Figure 5G). As the stimulation intensity of the RVS CR increases, the post-stimulus cross-trial phase distribution displays a feature characteristic of a phase reset: irrespective of the pre-stimulus phase at stimulation onset, the stimulus resets the phase to a preferred value (Figures 5H,I). The pre-stimulus resetting index *E* from Equation 14 is close to zero irrespective of the stimulation intensity, whereas the post-stimulus resetting index increases more for increasing stimulation intensities (Figure 5J). The SVS CR stimulation shows a different phenomenon. For sufficiently strong intensities the phase of the stimulated subgroup gets increasingly, and finally tightly stimulus-locked, i.e., entrained across trials, throughout the entire stimulus-locked time window (Figures 5L,M). Accordingly, the resetting index does not show a difference between the pre- and the post-stimulus period (Figure 5N). Obviously, the SVS CR stimulation results in an entrainment of the subgroup to the SVS CR stimulation signal. The other three neuronal subgroups show similar effects (results not shown).

**Figure 6:**
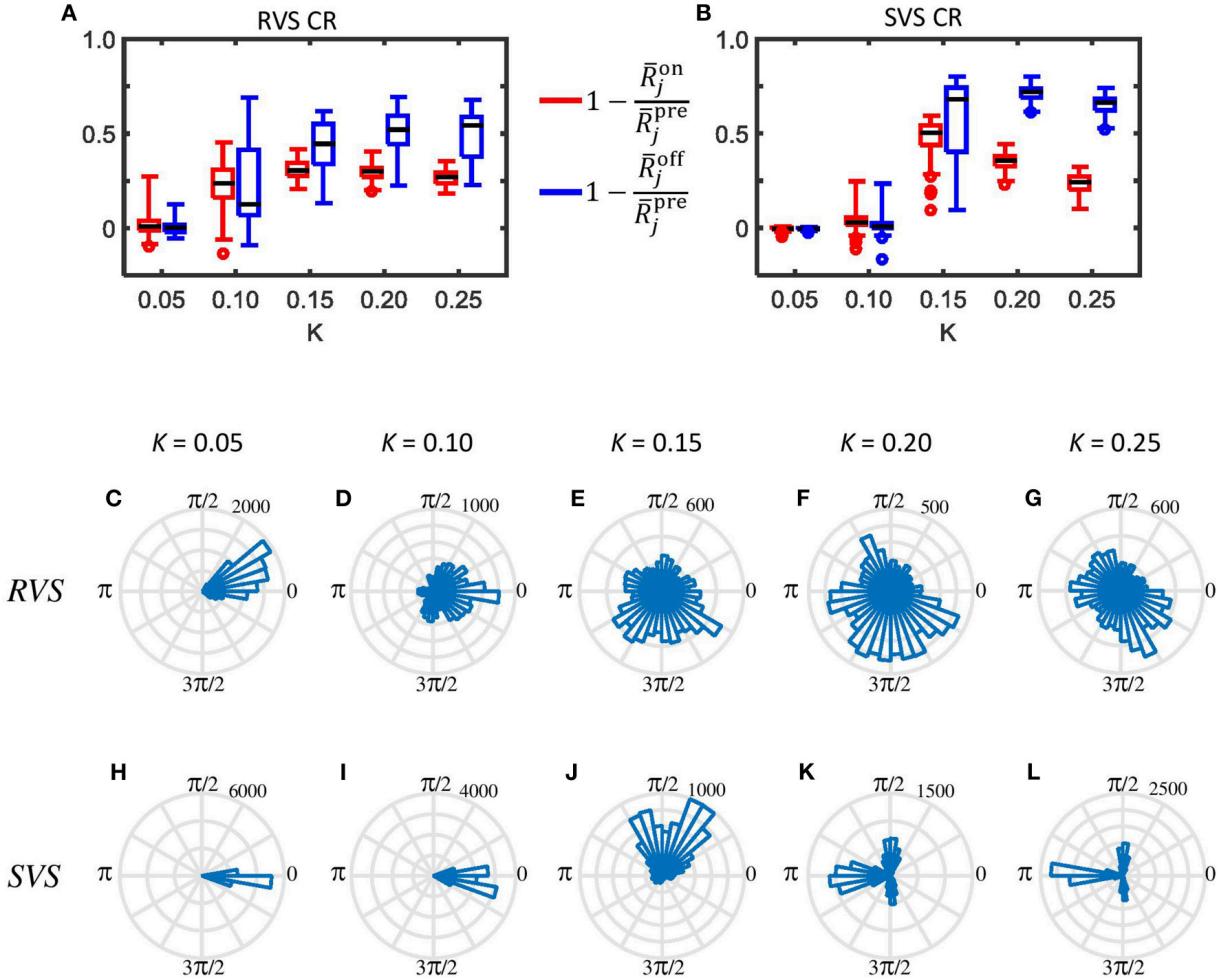
**Typical examples of the desynchronizing effect of the RVS and SVS CR stimulation on the mesoscopic level. (A)** Boxplots (*n* = 44 samples) show the distributions of the acute CR effect (Equation 17) in red and of the acute after-effect (Equation 18) in blue, induced in all subgroups of all eleven networks by the RVS CR stimulation at different stimulation intensities *K*. The CR-on period lasts for only 64 s. **(B)** as **(A)** for the SVS CR stimulation. **(C–G)** Angular histogram plots of the phase differences ϑ_23_ between subgroups 2 and 3 for different stimulation intensities K. Each plot shows the distribution of the 10.000 phase differences ϑ_23_, obtained during the last 10 s of the 64 s lasting RVS CR-on period (*n* = 10.000 phase difference samples). **(H–L)** as **(C–G)** for the SVS CR stimulation within the same initial network.

Since RVS and SVS CR affect the subgroups differently, we analyze the desynchronization of all subgroups by CR also for different stimulation intensities. Figure 6A illustrates that the acute RVS CR effect on the subgroups of all eleven initial networks as well as the acute after-effect are present already for *K* = 0.10. The acute effect of RVS CR peaks for *K* = 0.15 and slightly decreases for higher intensities. The acute after-effect of RVS CR builds up and saturates with increasing intensity. In contrast, for the SVS CR stimulation an acute effect and acute after-effect shows up only at *K* = 0.15. With increasing intensity the acute effect of SVS CR further decreases, whereas the acute after-effect peaks at *K* = 0.20 and slightly decreases above (Figure 6B). Another difference between the RVS and SVS CR stimulation is that for *K* = 0.20 and 0.25 the differences between the acute CR effect and the acute after-effect are larger for the SVS CR (Figure 6B).

In order to study the effect of CR stimulation on the phase-shifts between the different subgroups within the networks, we determined the phase differences between subgroup 2 and 3 for each ms during the last 10 s of the RVS as well as the SVS CR stimulation at different *K*-values in one network. Without stimulation the average and standard deviation of all 10,000 values of the phase difference ϑ_23_ is 6.2379±0.0008 rad (results not shown). Accordingly, the corresponding circular distribution of the phase difference ϑ_23_ has one prominent peak (where all values are comprised in one bin when displayed as in Figure 6, data not shown). The angular phase difference histograms in Figures 6C–G illustrate that the RVS CR induces a certain jitter of the phase differences ϑ_23_ already for *K* = 0.05, and that the distribution is not homogeneous for any shown *K*-value. The SVS CR induces also some jitter in ϑ_23_ at *K* = 0.05 (Figure 6H) but less than the RVS CR does (Figure 6C). Increasing the strength of *K* for the SVS CR (Figures 6H–L) induces a different pattern for ϑ_23_ than for the RVS CR stimulation: the majority of phase bins has no or just a very little number of entries, and for *K* = 0.20 and 0.25 a clear *asymmetric 3-peak distribution* shows up. Interestingly, the circular distribution does not peak at 0, which indicates that the SVS CR stimulation actually causes a non-vanishing phase shift, predominantly at π∕2, π, and 3π∕2 rad. Note during the last 10 s of the 64 lasting SVS CR-on period, seven different sequences are applied and not just one. Accordingly, the network is subjected to different phase-shifted entrainment stimulation patterns which, in turn, gives rise to the different rectangularly aligned peaks. For the other ten networks and the other combinations of subgroups we found similar asymmetric 3-peaks distributions for *K* = 0.20 and *K* = 0.25, although the height of the peaks vary, due to the applied sequences during the last 10 s, but the position of the peaks are always at π∕2, π, and 3π∕2 rad (results not shown). For the phase differences between other subgroups we obtain very similar results (not shown).

**Figure 7:**
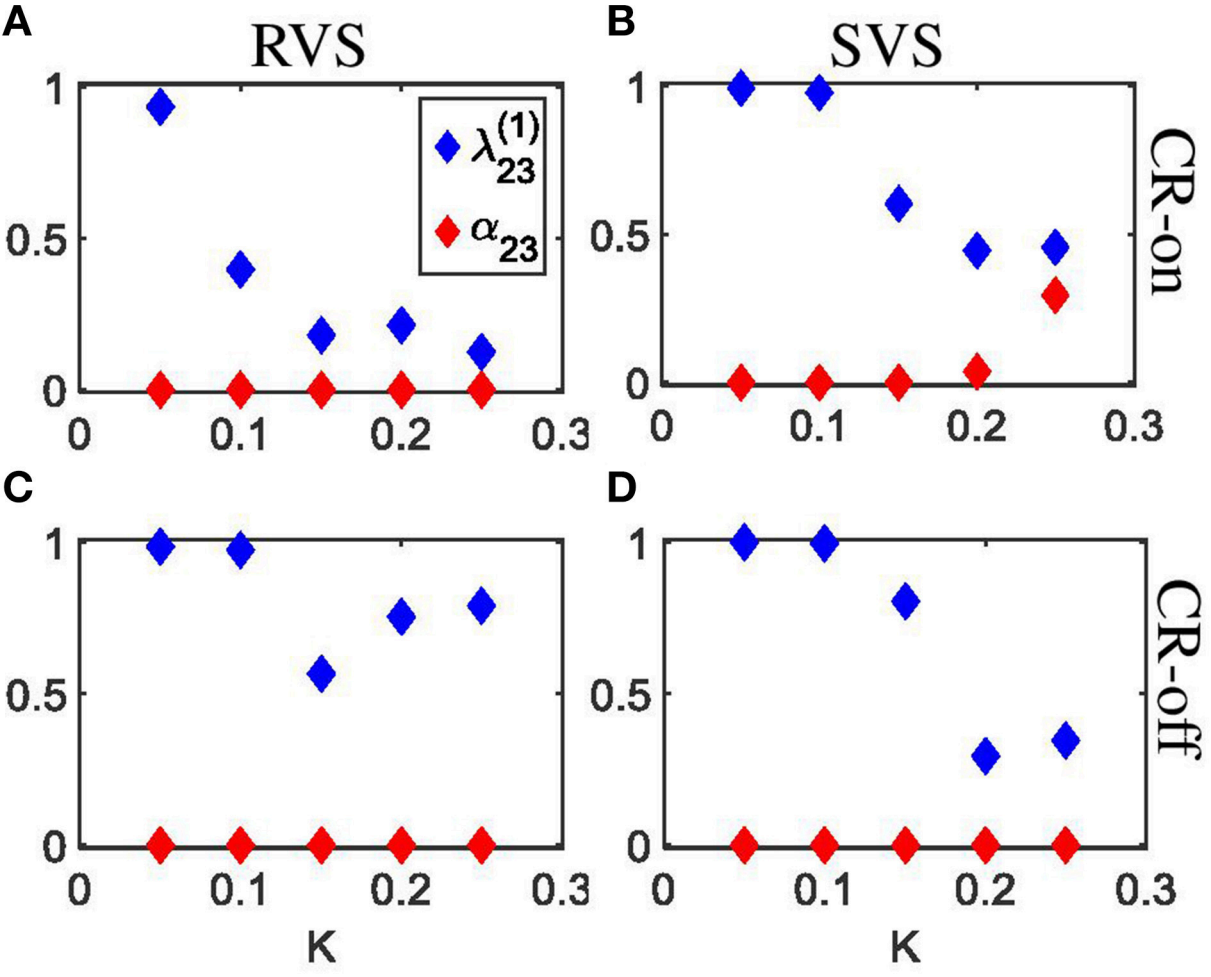
**Detection of rectangulary aligned peaks in the distribution of the phase differences between subgroups 2 and 3 during the last 10 s of the 64 s lasting CR-on period as well as for the first 10 s of the subsequent CR-off period as a function of the stimulation intensity K. (A)** The first order indices $\lambda_{23}^{\left(1\right)}$ (Equation 20) as well as the indices α_23_ (Equation 21) are determined for the last 10 s of the 64 s lasting RVS CR-on period as a function of the stimulation intensity K. **(B)** As **(A)** for the SVS CR stimulation. **(C)** As **(A)** for the first 10 s of the CR-off period. **(D)** As **(C)** for the SVS CR stimulation. The same initial network conditions are used for all panels.

The indices $\lambda_{23}^{\left(1\right)}$ from Equation 28 and α_23_ from Equation 29 shed further light on the stimulation-induced changes of the synchronization between subgroups 2 and 3. In accordance with the angular histogram plots from Figures 6C–G, RVS CR stimulation slightly reduces the phase synchronization between subgroups 2 and 3 and, hence, $\lambda_{23}^{\left(1\right)}\text{for }K=0.05$ (Figure 7A). For *K* = 0.1 and, in particular, for *K*>0.1 due RVS CR the phase synchronization between subgroups 2 and 3 is significantly reduced (Figure 7A). Only for *K*>0.1 the desynchronization between subgroups 2 and 3 persists during the acute OFF phase (Figure 7C). RVS CR stimulation does not cause 3-peak distributions (with peaks at π∕2, π and 3π∕2 rad) as detected with the index α_23_ from Equation 29. In contrast to RVS CR and in accordance with the corresponding angular histogram plots from Figures 6H–L, the phase synchronization between subgroups 2 and 3 and, hence, $\lambda_{23}^{\left(1\right)}$ hardly decreases for stimulation intensities of SVS CR up to *K* = 0.1. For *K* = 0.15 SVS CR stimulation causes a more pronounced reduction of the phase synchronization between subgroups 2 and 3 as evidenced by a reduced $\lambda_{23}^{\left(1\right)}$ with α_23_ from Equation 29 being still close to zero (Figure 7B). For higher intensities K 3-peak distributions (with peaks at π∕2, π and 3π∕2 rad) emerge and get more pronounced, so that α_23_ increasingly exceeds the zero line (Figure 7B). For *K*> 0.15 we observe pronounced a desynchronization between subgroups 2 and 3 in the acute off phase following cessation of stimulation (Figure 7D). As indicated by the vanishing index α_23_, 3-peak distributions (with peaks at π∕2, π and 3π∕2 rad) are not detected after stimulation is turned off, irrespective of the stimulation intensity K (Figure 7D). Very similar results were obtained for the phase difference between the other subgroups (data not shown).

### Single- and two-stage CR stimulation with weak onset

**Figure 8:**
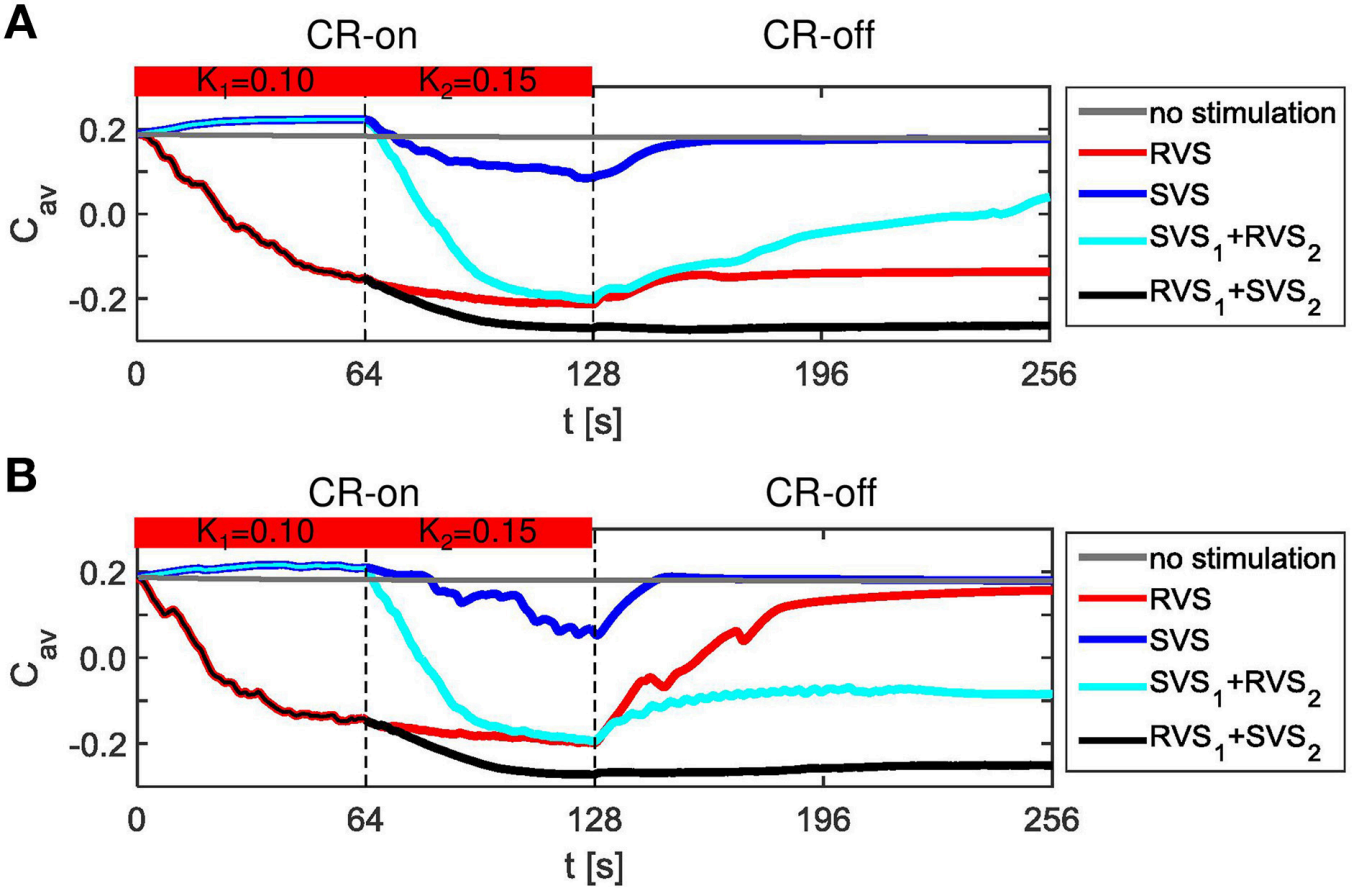
**Dynamics of *C*_*av*_ for the two-stage CR stimulation with weak onset. (A)** Results for the same initial network conditions and sequence orders as used in Figure 3. **(B)** Results obtained for other initial network conditions and sequence orders than used in **(A)**. The horizontal bar represents the two consecutive CR-on periods. During the first CR on period (0–64 s) the stimulation intensity equals *K*_*1*_ = 0.10, during the second CR-on period (64–128 s) *K*_*2*_ = 0.15, except for the no stimulation condition for which *K*_*1*_ = *K*_*2*_ = 0.00. The dashed vertical lines indicate the end of a CR-on period. No stimulation signals are delivered during the CR-off period (128–256 s) and *C*_*av*_ evolves spontaneously.

RVS CR stimulation is already effective at weak stimulation intensities, whereas SVS CR stimulation requires higher intensities to unfold its superior efficacy. We specifically hypothesize that RVS CR stimulation might render the network more susceptible to SVS CR stimulation by inducing a preparatory desynchronization. In this way SVS CR stimulation might be even more effective and require shorter stimulation durations. To study whether this approach is actually specific, from a more general perspective, we study whether CR stimulation can be empowered by a preparatory CR stimulation, of RVS or SVS type, at weaker intensities. To investigate this idea, the first CR stimulation is applied with a stimulation intensity *K*_*1*_ = 0.10 and the second CR stimulation is applied with a stimulation intensity *K*_*2*_ = 0.15. Since both CR approaches are applied to the same initial network as used in Figure 3 as well as with the same sequence orders as used in Figures 3A,B, the effect of the preceding CR approach with weaker onset can be determined. The obtained results of the different CR combinations show that all combinations do influence the average synaptic weight *C*_*av*_ and suggest that *C*_*av*_ is reduced in two cases (Figure 8A). The strongest reduction is obtained for the two-stage CR-stimulation with weak onset and RVS_1_ and SVS_2_. This results in a smaller final *C*_*av*_-value compared to the short single-stage SVS CR stimulation with intensity *K* = 0.15 as shown in Figure 3B. Also the long single-stage RVS CR-stimulation with weak onset (Figure 8A) induces a smaller final *C*_*av*_-value than the short single-stage RVS CR-stimulation with intensity *K* = 0.15 as shown in Figure 3A. A reduction of the long-lasting desynchronizing effect is observed for the long single-stage SVS CR-stimulation (compare Figure 8A with Figure 3B for *K* = 0.15). Obviously the different network conditions at the start of the 64 s lasting SVS CR-on period with *K* = *K*_*2*_ = 0.15 have a relevant effect on the stimulation outcome. The less advantageous network conditions at the start of the second SVS CR-on period (*K*_*2*_ = 0.15) were caused by the preceding SVS CR-on period with *K*_*1*_ = 0.10. This shows that a too weak stimulation intensity of the first SVS CR stimulation can, in fact, be unfavorable for the second SVS CR stimulation with a stronger stimulation.

**Figure 9:**
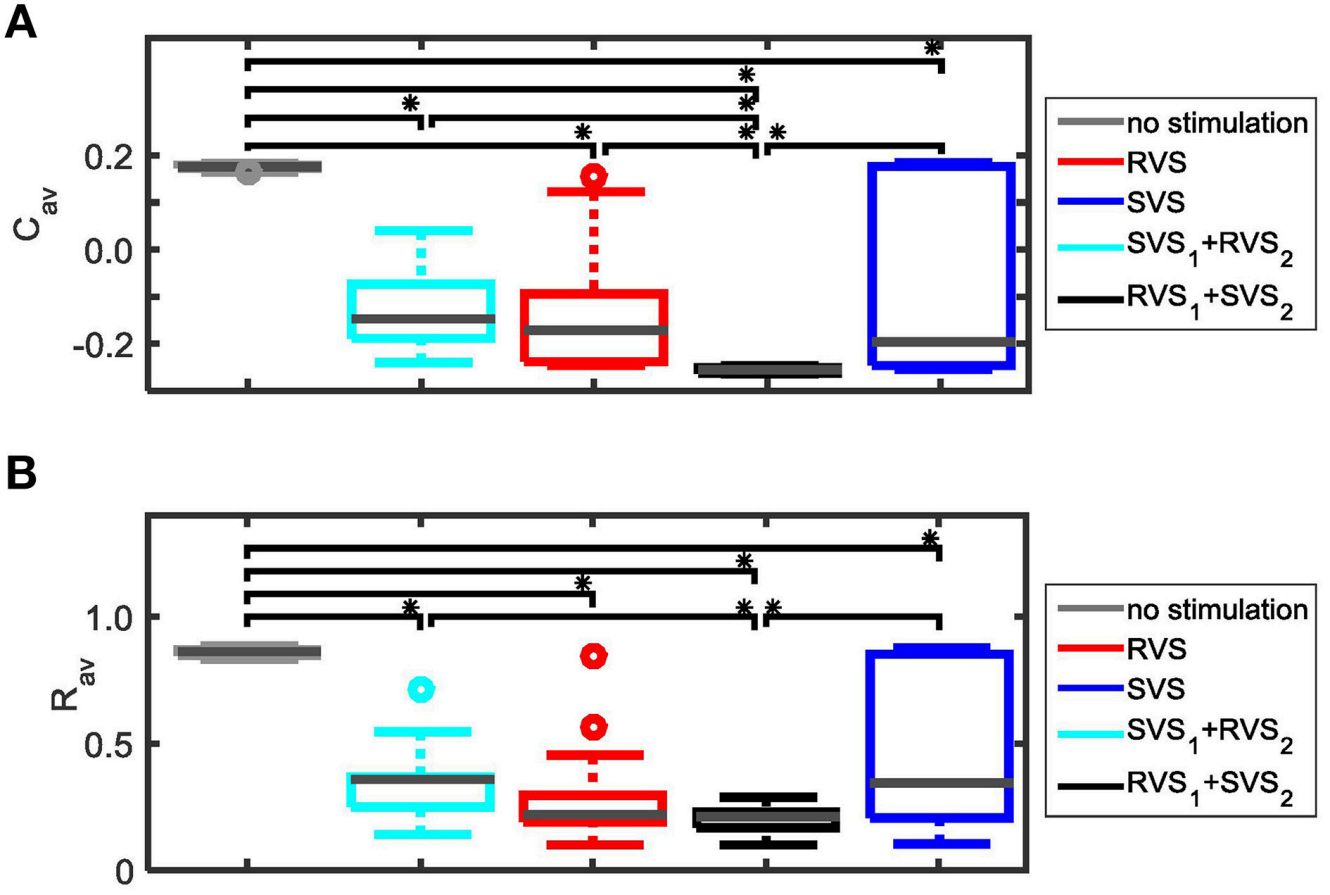
**Comparison of anti-kindling effects of the CR-stimulations with weak onset. (A)** Boxplots of the average synaptic weights, *C*_*av*_, at *t* = 256 s for the four possible combinations of the two CR approaches (*K*_*1*_ = 0.10, *K*_*2*_ = 0.15) and the control stimulation (*K*_*1*_ = *K*_*2*_ = 0.00). **(B)** As **(A)** for the average order parameter, *R*_*av*_, at *t* = 256 s. For each combination of CR approaches eleven samples were used. The gray horizontal line within the box represents the median, the box itself the middle 50% and the whiskers below (above) the box the first (last, respectively) 25%. Outliers are defined as 1.5 times the length of the box below or above the box and represented by open circles. An asterisk indicates a statistically significant lower *C*_*av*_- or *R*_*av*_-value (one-sided Mann-Whitney test, *p* < 0.05).

For the initial network and sequence orders as used in Figures 3, 8A, the two-stage CR-stimulation (RVS_1_, SVS_2_) with weak onset causes the strongest reduction of the *C*_*av*_-value. The second strongest reduction is achieved by the single-stage RVS CR-stimulation with weak onset (Figure 8A). However, different initial network conditions or other sequence orders can lead to different results (Figure 8B). Therefore, all simulations are executed for eleven samples. All two-stage CR-stimulation (RVS_1_, SVS_2_) trials with weak onset revealed the most favorable results (one-sided Mann-Whitney test, *p* < 0.05; Figure 9). The two-stage CR-stimulation (RVS_1_, SVS_2_) with weak onset causes the strongest reduction of *C*_*av*_-values for (*K*_*1*_, *K*_*2*_) = (0.10, 0.15), and the difference with the other combinations is statistically significant (one-sided Mann-Whitney test, *p* < 0.05), (Figure 9A). The resulting final *R*_*av*_ distributions are statistically not different for the two-stage CR stimulation and single-stage RVS CR-stimulation with weak onset. However, *R*_*av*_ caused by the two-stage CR stimulation with RVS_1_ and SVS_2_ is smaller than the other two combinations of the CR-approaches and the difference is statistically significant (one-sided Mann-Whitney test, *p* < 0.05; Figure 9B).

**Figure 10:**
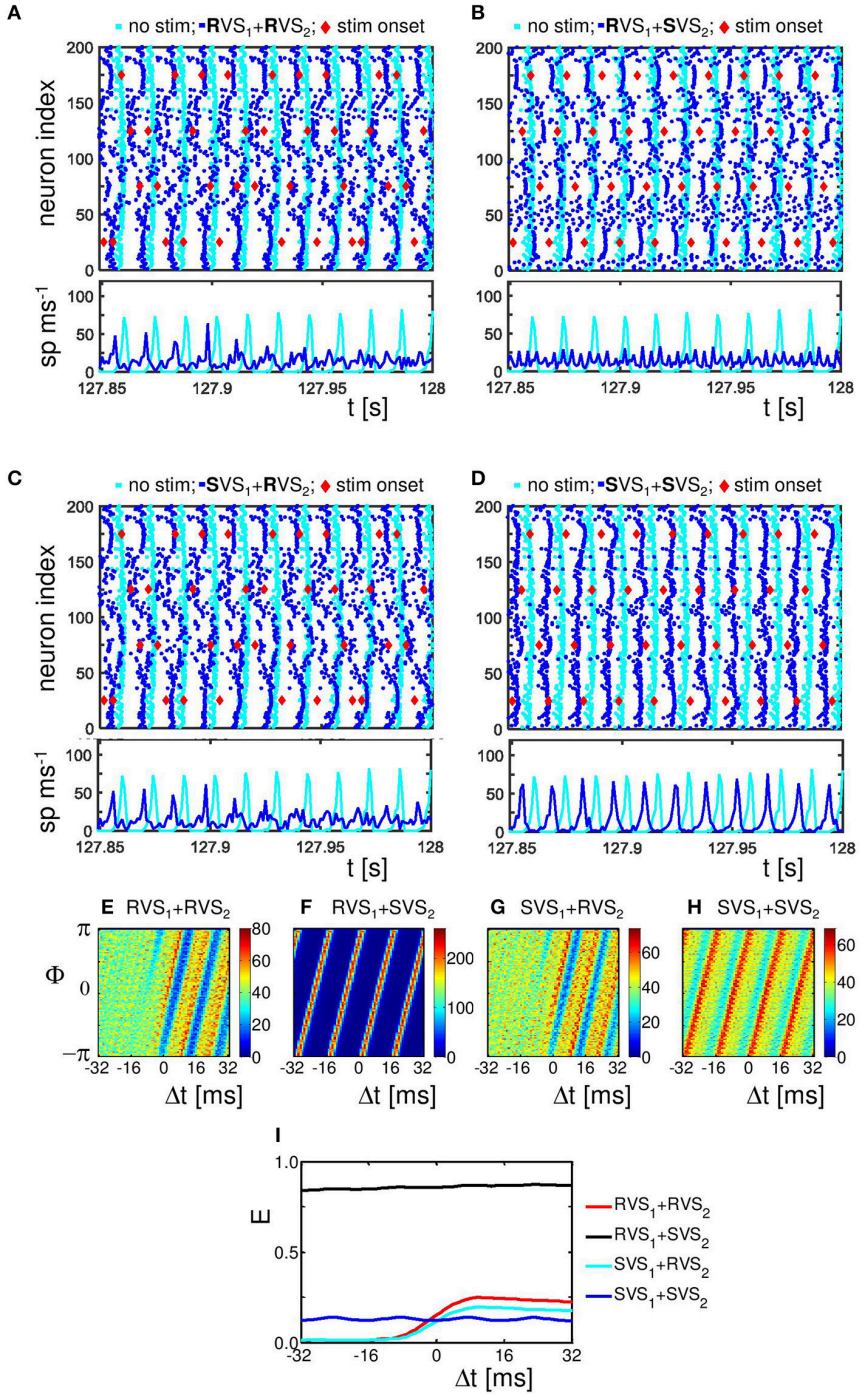
**Effect of single-and two-stage RVS and SVS CR stimulations with weak onset on the spike times at the end of the second 64 s lasting CR-on period. (A)** Raster plot and spike counts of the last 150 ms of the second CR-on period for the single-stage RVS CR stimulation (*K*_*1*_ = 0.10, *K*_*2*_ = 0.15; blue dots) and for the control stimulation with *K* = 0.00 (cyan dots). The stimulation onsets are represented in all panels by red diamonds. The spike counts are determined for each time bin of 1 ms. **(B)** as **(A)** for the two-stage (RVS_1_, SVS_2_), CR (*K*_*1*_ = 0.10, *K*_*2*_ = 0.15; blue dots). **(C)** As in **(A)** with blue dots for the spike times of the two-stage (SVS_1_, RVS_2_), CR (*K*_*1*_ = 0.10, *K*_*2*_ = 0.15). **(D)** As in **(B)** with blue dots for the spike times of the single-stage SVS CR (*K*_*1*_ = 0.10, *K*_*2*_ = 0.15) **(E, G)** Distributions of the mean phase of neurons 51–100, Φ_sg = 2_(Δt), averaged across the 4000 activation times of stimulation site 2 (= neuron 75) within the RVS_2_ CR-on period under the condition that first RVS_1_ **(E)** or SVS_1_ **(G)** was applied. **(F, H)** as **(E, G)** within the SVS2 CR-on period for RVS_1_ **(F)** and SVS_1_ **(H)**. **(I)** Resetting index *E*(Δ*t*) determined for the second CR-on period for the different combinations of RVS and SVS CR stimulations with *K*_*1*_ = 0.10 and *K*_*2*_ = 0.15. For all panels are the same initial network conditions and sequence orders as used in Figure 3.

Figure 10 illustrates that the RVS CR is not so sensitive to different initial conditions as the SVS CR stimulation (*K*_*1*_ = 0.10 and *K*_*2*_ = 0.15). The second CR-on periods of Figures 10A,B started with spike timing patterns as shown in Figure 5C, those from Figures 10C,D with spike timing patterns as in Figure 5D. Despite these differences applying a RVS CR during the second CR-on period results in similar raster plots and spike counts (Figures 10A,C). If a SVS CR stimulation is applied during the second CR-on period, the different conditions at the beginning of this CR-on period will result in different raster plots and spike counts (Figures 10B,D). It seems that a slightly reduced synchronization, induced by the RVS_1_ CR, makes it possible for the SVS_2_ CR stimulation to desynchronize the neuronal population, where the phase of the (sub)group of neurons gets time locked to the stimulation onsets. The distributions of the mean phase of neuronal subgroup 2, Φ_*sg* = 2_(△*t*) during the second CR-on period display patterns characteristic of phase resets for the RVS_2_ and of phase entrainment for the SVS_2_ CR stimulation (Figures 10E–H). The final phase reset is stronger if first RVS_1_ is applied compared to SVS_1_ CR stimulation (Figure 10I). The entrainment structure of the cross-trial phase distribution during the SVS_2_ CR-on period is much more pronounced if RVS is applied during the first CR-on period compared to a SVS_1_ CR stimulation (Figure 10I). So, the SVS_2_ CR stimulation is much more sensitive to the (change in) initial conditions at the beginning of the second CR-on period than the RVS_2_ CR stimulation and can obtain a resetting index comparable with the short (64 s lasting) single-stage SVS CR stimulations with a stronger stimulation intensity (compare Figures 5N at *K* = 0.20 with Figure 10I at *K*_*2*_ = 0.15).

**Figure 11:**
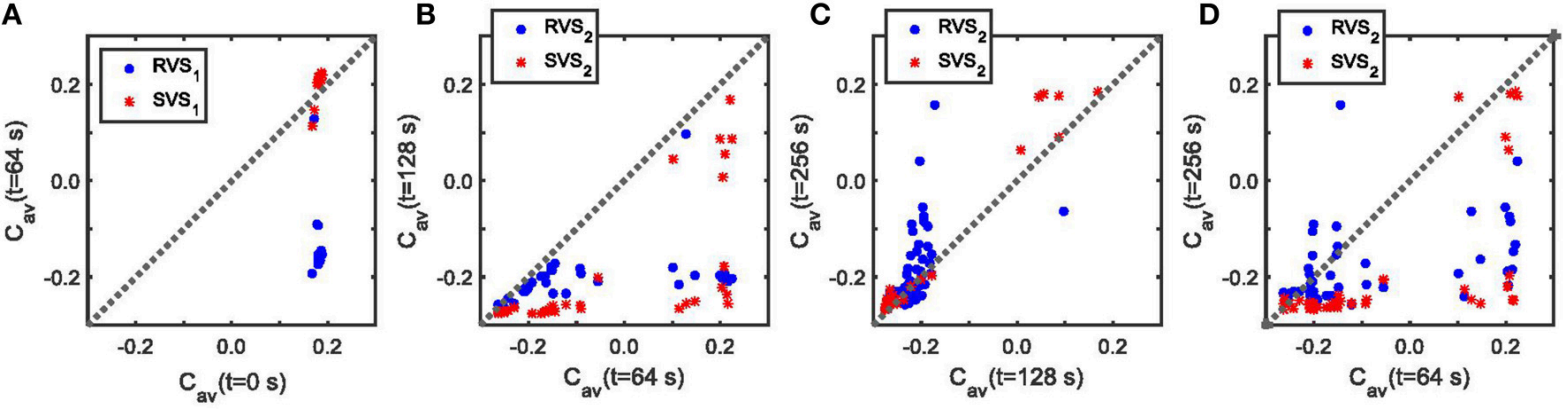
**The effect of the RVS and SVS CR-stimulations for weak stimulation intensities, *K*, on the average synaptic connectivity. (A)** *C*_*av*_ at the end of the first CR-on period vs. *C*_*av*_ at the beginning of the first CR-on period for RVS_1_ (red asterisks) and SVS_1_ (blue circles) CR-stimulation signals with *K*_*1*_ = 0.10. **(B)** *C*_*av*_ at the end of the second CR-on period vs. *C*_*av*_ at the beginning of the second CR-on period under the condition that RVS_2_ (red asterisks) or SVS_2_ (blue circles) CR-stimulation signals are delivered during the second CR-on period with stimulation intensity *K*_*2*_ = 0.15. **(C)** *C*_*av*_ at the end of the CR-off period vs. *C*_*av*_ at the end of the second CR-on period under the condition that RVS_2_ (red asterisks) or SVS_2_ (blue circles) CR-stimulation signals are delivered during the second CR-on period with *K*_*2*_ = 0.15. **(D)** *C*_*av*_ at the end of the CR-off period vs. *C*_*av*_ at the beginning of the second CR-on period under the condition that RVS_2_ (red asterisks) or SVS_2_ (blue circles) CR-stimulation signals with *K*_*2*_ = 0.15 were delivered during the second CR-on period.

Figure 11 sheds some light on the effects of the two-stage CR with weak onset on the average synaptic weight C_*av*_. For a very weak stimulation intensity (*K*_*1*_ = 0.10) during the first CR-on period, the SVS_1_ CR-stimulation is not able to decrease the average synaptic strength, *C*_*av*_ (Figure 11A). But a RVS_1_ CR-stimulation with *K*_*1*_ = 0.10 decreases the initial *C*_*av*_ value (Figure 11A). The effectiveness of reducing *C*_*av*_ during the second CR-on period with *K*_*2*_ = 0.15 depends on the *C*_*av*_ -value at the onset of this second CR-on period, especially the SVS_2_ CR-stimulation can result in a large range of *C*_*av*_ -values for initially large *C*_*av*_-values (*C*_*av*_ > 0.1; Figure 11B). However, for initially small *C*_*av*_ -values it induces a smaller *C*_*av*_ than the RVS_2_ CR-stimulation (Figure 11B) and also has a superior aftereffect on the average synaptic weight during the CR-off period (Figure 11C). Except for large *C*_*av*_-values at the onset of the SVS_2_ CR-stimulation, the SVS_2_ CR-stimulation with *K*_*2*_ = 0.15 induces a very robust and long-lasting reduction of the *C*_*av*_-values (Figure 11D). The equivalent for the RVS_2_ CR stimulation is less effective and less robust (Figure 11D).

In summary, for the two-stage CR-stimulation with weak onset (*K*_*1*_ = 0.10; *K*_*2*_ = 0.15) it is best to first apply the RVS CR and then the SVS CR-stimulation because the RVS_1_ CR-stimulation with *K*_*1*_ = 0.10 reduces the amount of synchronization of the initially very strongly synchronized neuronal activity (Figure 5C) as well as the very strong *C*_*av*_ (Figure 11A), and the SVS_2_ CR stimulation is robustly and effectively performing for *C*_*av*_ ≤ 0.1 (Figure 11D) as well as entraining the mean phases of the neuronal subpopulations to the stimulation onsets (Figures 10F,I).

**Figure 12:**
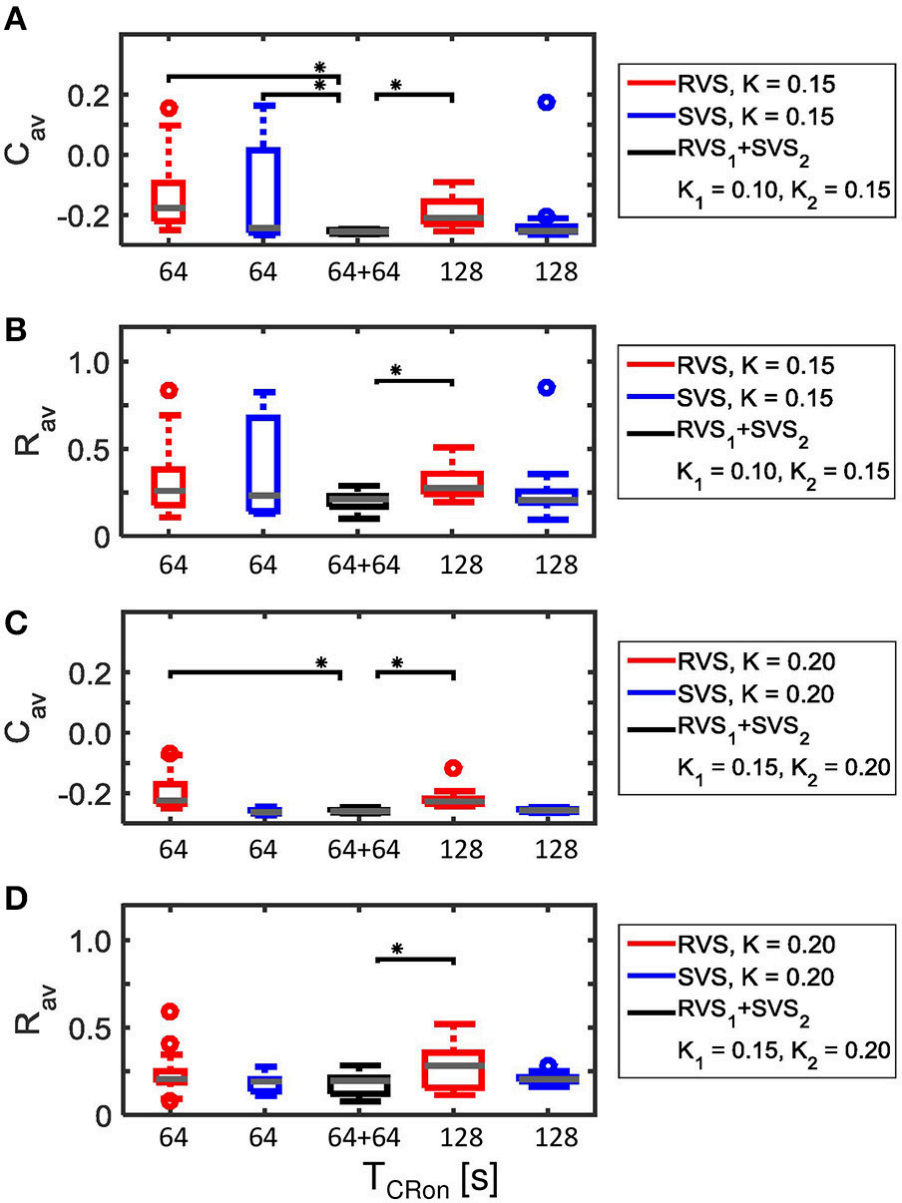
**Comparison of anti-kindling effects of four single-stage CR-stimulations with one two-stage CR-stimulation with weaker onset stimulation intensity (*K*_*1*_ < *K*_*2*_). (A)** Boxplots of *C*_*av*_(*t* = end of CR-off period) for different CR-stimulation approaches with *K* = 0.15 for the single-stage CR-stimulations. The CR-off period lasts as long as the total CR-on period. From the left to the right: 64 s lasting RVS CR, 64 s lasting SVS CR, two-stage CR stimulation with RVS_1_ CR with *K*_*1*_ = 0.10 and SVS_2_ CR with *K*_*2*_ = 0.15 and finally a 128 s lasting CR-off period, 128 s lasting RVS CR, 128 s lasting SVS CR. **(B)** as **(A)** for *R*_*av*_(*t* = end of CR-off period). **(C)** as **(A)** for *K* = 0.20 and the middle boxplot represent the results of the two-stage CR-stimulation with *K*_*1*_ = 0.15 and *K*_*2*_ = 0.20.**(D)** as **(B)** for *K* = 0.20 and the middle boxplot represent the results of the two-stage CR-stimulation with *K*_*1*_ = 0.15 and *K*_*2*_ = 0.20. For each condition (*K* value, CR approach and duration) eleven samples are used. The gray horizontal line within the box represents the median, the box itself the middle 50% and the whiskers below (above) the box the first (last, respectively) 25%. Outliers are defined as 1.5 times the length of the box below or above the box and represented by open circles. An asterisk indicates a statistically significant lower *C*_*av*_- or *R*_*av*_-value (one-sided Mann-Whitney test, *p* < 0.05).

This two-stage CR-stimulation (RVS_1_ with *K*_*1*_ = 0.10 and SVS_2_ with *K*_*2*_ = 0.15) performs better on *C*_*av*_ than the short as well as the long single-stage RVS CR-stimulation with *K* = 0.15 and also better than the short single-stage SVS CR-stimulation with *K* = 0.15 (Figure 12A). The differences are statistically significant (one-sided Mann-Whitney-U-Test, *p* < 0.05). Although this optimal two-stage CR-stimulation is statistically not different from the long single-stage SVS CR-stimulation with *K* = 0.15, it has the advantage that it uses a smaller stimulation intensity during the first CR-on period. The average order parameter, *R*_*av*_, also suggests that the two-stage CR-stimulation (RVS_*1*_ with *K*_*1*_ = 0.10 and SVS_2_ with *K*_*2*_ = 0.15) has a stronger desynchronizing effect in comparison with the other CR approaches (Figure 12B). Increasing the stimulation intensities for the two-stage CR-stimulation (RVS_1_ with *K*_*1*_ = 0.15 and SVS_2_ with *K*_*2*_ = 0.20) shows similar effects Figures 12C,D).

**Figure 13:**
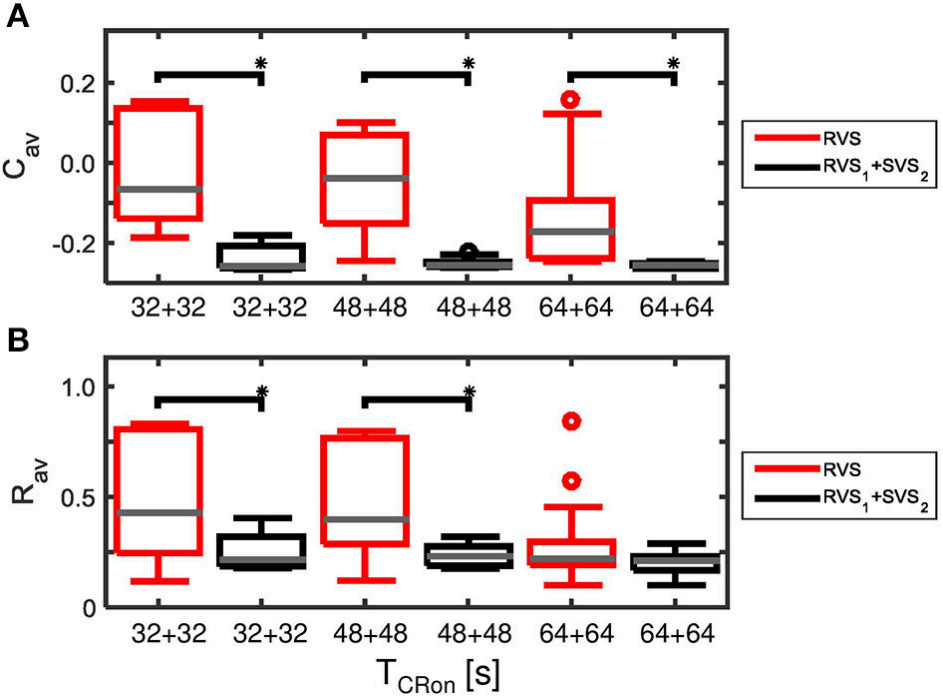
**Comparison of anti-kindling effects induced by the two-stage CR and the single-stage RVS CR stimulation, both with weak onset intensity (*K*_*1*_ = 0.10, *K*_*2*_ = 0.15), as a function of the duration of the total CR-on period, *T*_*CR*−*on*_. (A)** Boxplots of *C*_*av*_(*t* = end of CR-off period) for different lasting CR-on periods. **(B)** Boxplots of *R*_*av*_(*t* = end of CR-off period) different lasting CR-on periods. Results obtained by the two-stage (*RVS*_*1*_, *SVS*_*2*_) CR stimulation are shown in black, for the single-stage CR-stimulation in red. *K*_*1*_ = 0.10 and *K*_*2*_ = 0.15. The CR-off period lasts as long as the total CR-on period *T*_*CR*−*on*_. For each condition (CR approach and duration) eleven samples are used. The gray horizontal line within the box represents the median, the box itself the middle 50% and the whiskers below (above) the box the first (last, respectively) 25%. Outliers are defined as 1.5 times the length of the box below or above the box and represented by open circles. An asterisk indicates a statistically significant lower *C*_*av*_- or *R*_*av*_-value (one-sided Mann-Whitney test, *p* < 0.05).

The advantage of the two-stage approach becomes even more pronounced for particularly short stimulation durations. Decreasing the duration of *each* CR-on period to 32 s or 48 s results in more reduced *C*_*av*_ as well as *R*_*av*_ values at the end of the CR-off period for the two-stage CR-stimulation (RVS_1_ with *K*_*1*_ = 0.15 and SVS_2_ with *K*_*2*_ = 0.20) than for the single-stage RVS CR-stimulation with *K*_*1*_ = 0.10 and *K*_*2*_ = 0.15 with a total CR-on duration of 64 s or 96 s, respectively (Figure 13). This is because for *K* = 0.15 the SVS CR stimulation decreases *C*_*av*_ much faster than the RVS CR stimulation does. The mentioned reductions caused by decreasing the CR-on period are statistically significant (one-sided Mann-Whitney-U-Test, *p* < 0.05).

### Two-stage CR stimulation with constant stimulation intensity

So far the two-stage CR-stimulation with weak onset seems to be a favorable choice compared to the single-stage CR-stimulation in the range of *K* = *K*_*2*_ ∈ [0.15, 0.20]. In this section we will investigate the effect of the two-stage CR-stimulation with identical stimulation intensities. This is to study whether the two-stage CR approach might be applicable beyond the initial concept of a two-stage CR stimulation with weak onset.

**Figure 14:**
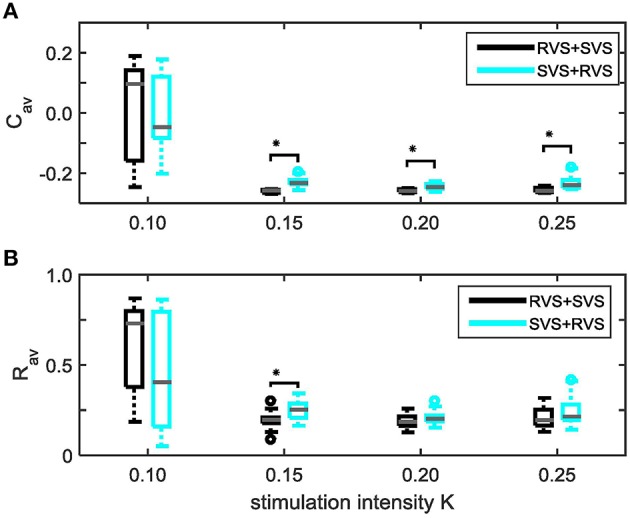
**Comparison of anti-kindling effects induced by the two different two-stage CR-stimulations as a function of constant stimulation intensity *K*_*1*_ = *K*_*2*_ = *K*. (A)** shows the boxplots of the *C*_*av*_ results in black for the two-stage CR-stimulation with RVS_1_ and SVS_2_, and in cyan for the two-stage CR-stimulation with SVS_1_ and RVS_2_, as a function of the constant stimulation intensity, *K*.**(B)** As **(A)** for the *R*_*av*_ results. Eleven samples are used for each condition (*K* value and order of the CR approaches). The distribution of the obtained *C*_*av*_ and *R*_*av*_ values are represented by a boxplot. The gray horizontal line within the box represents the median, the box itself the middle 50% and the whiskers below (above) the box the first (last, respectively) 25%. Outliers are defined as 1.5 times the length of the box below or above the box and represented by open circles. An asterisk indicates a statistically significant lower *C*_*av*_- or *R*_*av*_-value (one-sided Mann-Whitney test, *p* < 0.05).

Figure 14 shows that except for *K* = 0.10 the two-stage CR-stimulation with RVS_1_ and SVS_2_ CR causes a stronger reduction of *C*_*av*_-values than the two-stage CR with SVS_1_ and RVS_2_ CR, where the differences are statistically significant (one-sided Mann-Whitney-U-test, *p* < 0.05). However, for the very weak stimulation intensity of *K* = 0.10 the results are not very pronounced, and statistically there is no difference between the results obtained by the two different two-stage CR-stimulations. In what follows we will only compare the best two-stage CR stimulation variant (RVS_1_and SVS_2_) with the single-stage CR stimulations.

**Figure 15:**
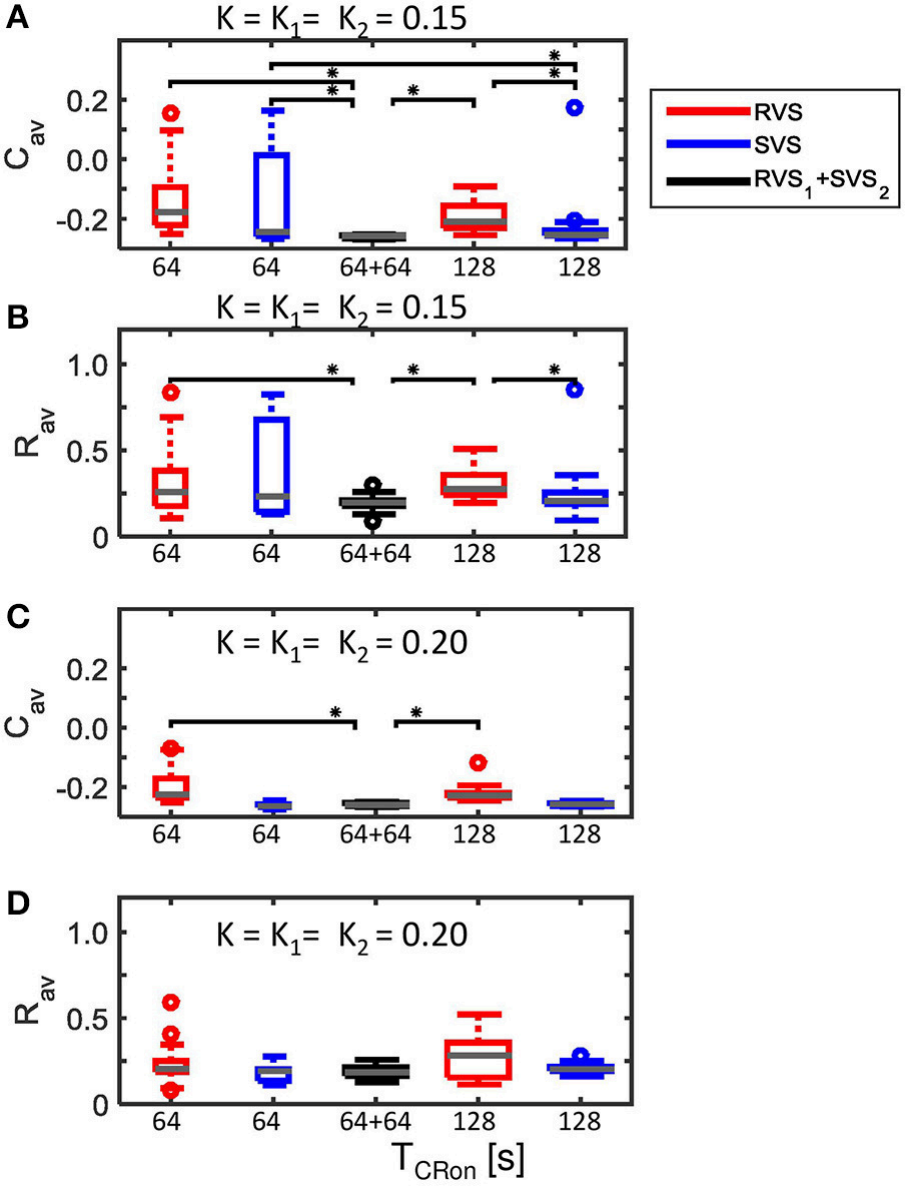
**Comparison of anti-kindling effects of four single-stage CR-stimulation with one two-stage CR-stimulation with constant stimulation intensity (*K*_*1*_ = *K*_*2*_). (A)** Boxplots of *C*_*av*_(*t* = end of CR-off period) for different CR-stimulation approaches with *K* = 0.15 for the single-stage CR-stimulations. The CR-off period lasts as long as the total CR-on period. From the left to the right: 64 s lasting RVS CR, 64 s lasting SVS CR, two-stage CR-stimulation with RVS_1_ and SVS_2_ CR-on period and finally a 128 s lasting CR-off period, 128 s lasting RVS CR, 128 s lasting SVS CR. **(B)** as **(A)** for *R*_*av*_(*t* = end of CR-off period). **(C)** as **(A)** for *K* = 0.20. **(D)** as **(B)** for *K* = 0.20. Eleven samples are used for each boxplot. The gray horizontal line within the box represents the median, the box itself the middle 50% and the whiskers below (above) the box the first (last, respectively) 25%. Outliers are defined as 1.5 times the length of the box below or above the box and represented by open circles. An asterisk indicates a statistically significant lower *C*_*av*_- or *R*_*av*_-value (one-sided Mann-Whitney test, *p* < 0.05).

Figure 4 showed that for *K* = 0.15 the average synaptic weight, *C*_*av*_, at the end of the CR-off period is not very robust against initial network conditions and sequence orders. Extending the total CR-on period from 64 to 128 s increases the robustness (compare the two boxplots at the left to the two boxplots at the right side of Figure 15A). The *C*_*av*_-value resulting from the most effective two-stage CR-stimulation for *K* = 0.15 (RVS_1_and SVS_2_) is smaller and more robust against initial network conditions and sequence orders than the short and long single-stage RVS and the short single-stage SVS CR stimulation (Figure 15A). These differences are statistically significant (one-sided Mann-Whitney-U-test, *p* < 0.05). The median order parameter, averaged over the last 1.6 s of the CR-off period, *R*_*av*_, is smaller for the two-stage CR stimulation than for the single stage CR stimulations, however, the difference is only statistically significant for the single-stage RVS CR stimulations, independent of the duration of the single-stage CR stimulation (Figure 15B). For stronger stimulation intensities (*K* = 0.20 and *K* = 0.25) the advantage of using the two-stage CR-stimulation (RVS_1_and SVS_2_) is less pronounced, but still statistically not worse than the single-stage SVS CR-stimulation (for *K* = 0.20 see Figures 15C,D; for *K* = 0.25 results are similar and therefore not shown), although the short (64 s) single-stage SVS CR-stimulation should be preferable because of its shorter stimulation duration.

## Discussion

In this study we investigated if a two-stage CR-stimulation induces better anti-kindling effects than a single-stage CR-stimulation for weak to intermediate stimulation intensities (*K* ≤ 0.25). The most optimal CR-approach would be the one that induces the best long-lasting anti-kindling results for the shortest and weakest CR-stimulation. The main result of this study is that the two-stage CR-stimulation with first a RVS CR-stimulation with intensity *K*_*1*_ = 0.10, followed by a SVS CR-stimulation with intensity *K*_*2*_ = 0.15 is the optimal CR-approach for weak stimulation intensities (Figure 12), even for short CR-stimulations (Figure 13). This is because it uses the advantage of a very weak RVS CR-stimulation to reduce the average synaptic strength, *C*_*av*_, and the advantage of the weak SVS CR-stimulation of inducing stronger and more robust anti-kindling effects. For weaker *K*_*2*_ values the SVS CR-stimulation cannot induce stronger anti-kindling effects (Figure 14) than the short single-stage RVS CR-stimulation (Figure 4). Therefore, the short single-stage RVS CR-stimulation is preferred if no stronger stimulation intensity can be used than *K*≥0.10. For stronger stimulation intensities (*K* = *K*_*1*_ = 0.20) the two-stage CR-stimulation induces statistically similar anti-kindling effects as the short single-stage SVS CR-stimulation (Figures 15C,D), but because of the shorter duration the single-stage SVS CR-stimulation is preferred in case no weaker stimulation intensities can be used than *K* = 0.20.

From a previous study (Zeitler and Tass, 2015) with single-stage CR stimulation it is known that for very weak stimulation intensity (*K* = 0.10) only the RVS CR-stimulation could induce small anti-kindling effects. It showed further that for intermediate stimulation intensities (0.20 ≤ *K* ≤ 0.50) the SVS CR-stimulation is the most optimal CR-approach. In that study we applied after three stimulation ON-cycles two stimulation OFF-cycles during the CR-on period. In the present study we used no stimulation OFF-cycles during the CR on-period(s), because preliminary results showed that without stimulation OFF-cycles the anti-kindling effects are increased, mainly for the very weak and weak RVS CR-stimulation, but also for the weak SVS CR stimulation (results not shown). The reason for this might be that the CR stimulation, without stimulation OFF-cycles as used in the current study, contains about 67% more ON-cycles. To investigate this we performed additional simulations in which we applied two stimulation OFF-cycles after each group of three simulation-ON cycles until in total 6000 stimulation ON-cycles were applied. The resulting CR-on period lasts 120 s and corresponds to a CR-on period of 96 s in case no stimulation OFF-cycles were applied. These additional simulations showed that if the two-stage CR-stimulation with weak onset contains the same amount of ON-cycles, similar anti-kindling effects are induced by the stimulation signals with ON:OFF ratios of 3:2 and 5:0, but for a ratio of 3:2 slightly stronger stimulation intensities (*K*_*1*_ = 0.13 and *K*_*2*_ = 0.16) are needed than for a ratio of 5:0 (*K*_*1*_ = 0.10 and *K*_*2*_ = 0.15; results not shown).

Lysyansky and coworkers showed in their computational model with phase oscillators that a permanent CR stimulation with high frequency bursts as stimulation signal induces desynchronization for weak stimulation intensities (Lysyansky et al., 2011). This is in good agreement with our finding that for a permanent CR stimulation with weak intensity a good desynchronization can be obtained.

As shown in numerous studies in neuroscience, psychology and education, learning effects can be reinforced by means of the spacing principle, i.e., by administering repeated stimuli spaced by pauses as opposed to administering a massed stimulus in a single long stimulation session (Ebbinghaus, 1913; Itoh et al., 1995; Frey and Morris, 1997; Menzel et al., 2001; Scharf et al., 2002; Cepeda et al., 2006, 2009; Pavlik and Anderson, 2008; Xue et al., 2011; Naqib et al., 2012). By the same token, as demonstrated computationally the spacing principle may also boost the CR-induced unlearning of abnormal synaptic connectivity and, hence, of abnormal neuronal synchrony (Popovych et al., 2015). For this demonstration, CR stimulation was applied at particularly weak intensities, so that neither acute effects (i.e., desynchronization under stimulation) nor long-lasting effects (i.e., anti-kindling) could be elicited with permanently delivered CR. Intriguingly, spaced CR stimulation at these particularly weak intensities can effectively induce anti-kindling. The dynamical mechanism behind this stimulation protocol is that the spaced stimulation causes the neuronal population to consecutively bounce from one attractor to another one, ultimately approaching desired attractors characterized by down-regulated synaptic connectivity and synchrony. The downside to this approach is the immense time it takes to build up anti-kindling effects (Popovych et al., 2015). For implanted stimulation treatments this might be tolerable. However, for non-invasive treatments, requiring compliance, e.g., requiring the patient to wear a stimulation device, it is indispensable to minimize treatment duration. In contrast, the two-stage CR stimulation is effective even at particularly short stimulation durations (Figure 13). Accordingly, one might use the spacing principle for single-stage CR stimulation at higher stimulation intensities. In addition, one could also apply the spacing principle to the two-stage CR stimulation in an attempt to further increase its efficacy.

The two-stage CR stimulation comprises two stimulation types, RVS and SVS CR stimulation, that both build up their effects on time scales, that are large compared to the period of the synchronized oscillation, and both act, in principle, in the same direction, i.e., they have desynchronizing and anti-kindling effects, at least for appropriate parameter ranges. Combining the two CR variants enables to induce anti-kindling at particularly weak intensities. In contrast, in the field of desynchronizing stimulation protocols preparatory stimuli have been used in computational studies (in the absence of STDP) that act on the short time scale of the period of the synchronized oscillation and enable an acute desynchronization when repetitively delivered as part of a double pulse stimulation (Tass, 2001a,b, 2002a,b), but typically have synchronizing effects if solely applied. The first, preparatory stimulus of a double pulse is stronger and causes a reset (and often a synchronization) of the collective dynamics within one (Tass, 2001a,b) or a few cycles of the collective oscillation, irrespective of the network's initial dynamic state (Tass, 2002a,b). The first stimulus might be a strong pulse (Tass, 2001a, 2002b) or a low-frequency pulse train (Tass, 2002a,b). The weaker, second stimulus is applied after a fixed time delay and hits the network in its vulnerable state in a stereotyped way, in this way causing a desynchronization. Although the two-stage CR stimulation and the double pulse stimulation share some conceptual similarities (empowered stimulation by combined stimulation protocols), both approaches are, in fact, quite different. In fact, it adds to the value of the two-stage CR approach that the preparatory type of stimulus, the RVS CR stimulation causes an acute desynchronization rather than a synchronization as compared to the first, strong and globally resetting stimulus of a double pulse.

Our analysis of the dynamics induced by RVS CR stimulation and SVS CR stimulation revealed clear differences (Figures 5–7). At sufficient intensities K RVS CR stimulation causes repetitive phase resets of the different neuronal subgroups (located close to the respective stimulation sites; Figure 5) along with a desynchronization between different subgroups (Figures 6, 7) as well as an overall desynchronization of the whole population (Figure 4) combined with a desynchronization on the mesoscopic level, i.e., of the different subgroups (Figure 6). In contrast, applied at sufficient intensity K, SVS CR stimulation induces a phase entrainment between subgroups and corresponding stimuli (Figure 5). Changing the sequence of the SVS CR stimulation gives rise to different phase shifted entrainment patterns and, hence, characteristic 3-peak distributions with peaks at π∕2, π and 3π∕2 rad (Figure 6) as detected by an appropriately designed index α_23_ from Equation 29 (Figure 7). The subgroups' mutually phase shifted entrainment is combined with a reduction of the overall synchronization (Figure 4) as well as of the synchronization on the mesoscopic level of subgroups (Figure 6). Combining a preparatory RVS CR stimulation at weak intensities (first stage) with a SVS CR stimulation at higher intensity (second stage) renders the latter more effective (Figures 9, 12) and furthers the SVS characteristic phase entrainment mechanism (Figure 10).

For our analysis of the stimulus-locking of the subgroups' phases we used the resetting index E_sg_(Δ*t*) for each subgroup *sg*, see Equation (16). This index is nothing but a circular mean of the phases at time Δt relative to the stimulus onsets. In case of a unimodal distribution of phase (i.e., periodic) variables, a circular mean is a standard measure (Batschelet, 1981). A quantity comparable to E_sg_(Δ*t*), based on a wavelet transformation and denoted as “phase-locking factor,” has been used to study phase resetting in EEG signals registered during sensory stimulation experiments (Tallon-Baudry et al., 1996; Makeig et al., 2002). However, since in all our simulations we only found phase distributions that were either close to uniform or unimodal, we used the circular mean for quantification. In contrast, multimodal distributions of the phase of an oscillatory signal may occur in the case of stimulus-locked response clustering (Tass, 2003d). The detection of the corresponding bi- or multimodal cross-trial distributions of the phases require different statistical methods (Tass, 2004). Applying a circular mean-based analysis to the phase difference of two oscillatory signals across trials led to quantities like the phase locking value (Lachaux et al., 1999) and the stimulus locked-phase synchronization index (Tass, 2003d).

A global pattern of intermittent bursting of synchronization was observed in the context of Parkinson's (Park et al., 2011). Accordingly, our stimulation approach could be tested in computational models showing that type of dynamics, or straight away in Parkinsonian monkeys or Parkinson's patients in comparison to standard CR approaches (see Tass et al., 2012a; Adamchic et al., 2014a). However, our approach is not limited to Parkinson's (see Introduction).

The dynamics of our model is quite complex due to STDP, and it is difficult to predict what the exact long-lasting anti-kindling effect is for different individual neuronal initial conditions as well as different sequence orders. Individually randomly varying background noise might even make it more complicated. Nevertheless, the impact of noise on stimulus responses is a relevant topic. For instance, it was shown that cross-trial response clustering in two coupled oscillators subjected to CR stimulation obeys a stochastic-like behavior (Tass, 2003c). In addition, in the presence of STDP noise may have unexpected, e.g., coupling- stabilizing effects (Popovych et al., 2013). Hence, further studies should address the impact of different noise processes on the outcome of different desynchronizing stimulation approaches.

In this paper, we investigated how the CR stimulation algorithm for weak stimulation intensities could be optimized by combining the RVS and the SVS CR approaches and by using permanent CR stimulation signals during the CR-on period. The results predict for weak onset better anti-kindling by the two-stage CR stimulation than by the single-stage RVS CR stimulation. Stimulation protocols of this kind might be tested for invasive as well as non-invasive CR approaches. In the case of acoustic CR stimulation for the treatment of tinnitus (Tass et al., 2012b) stimulation at particularly weak intensities might be beneficial for several reasons: In general, cognitive functions, communication and social interaction are less hindered by low-intensity sound therapy. In tinnitus with pronounced hearing loss two-stage CR might enable to reduce the stimulation intensity and, hence, reduce the risk of sound-induced hearing impairment. About 30–40% of the tinnitus patients additionally suffer from hyperacusis, an over-sensitivity to certain volume and frequency ranges of sound (Sheldrake et al., 2015). In such patients low-intensity sound therapy may prevent from unpleasant or painful perceptions. For invasive CR approaches the two-stage protocol might be advantageous, too. Standard high-frequency permanent deep brain stimulation for the treatment of Parkinson's disease (Benabid et al., 1991; Deuschl et al., 2006b) may cause side effects such as speech and gait deterioration (Mahlknecht et al., 2015). Some of these side effects might be caused by particular anatomical relations between targets and neighboring areas and/or fiber tracts passing by, others by sub-optimally positioned leads. In any case, the reduction of the amplitude of the stimulation current may help to reduce side effects. This computational study is intended to provide results that can be tested in pre-clinical (animal) studies and phase 1 (first in man) and subsequently phase 2 (proof of concept and dose finding) studies. Our model is qualitatively rather than biophysically realistic. Hence, our experimentally testable predictions are qualitatively and refer to, e.g., the strategy of two-stage stimulation and the related ratio between the stimulation intensity of the first vs. second stage—as opposed to absolute intensity values. A comparable qualitatively prediction of the ratio between the optimal intensity of standard high-frequency deep brain stimulation and an optimal intensity for CR deep brain stimulation was verified in a pre-clinical study in parkinsonian non-human primates and enabled to reveal pronounced long-lasting after-effects of CR stimulation (Tass et al., 2012b). By the same token, a dose finding study might reveal intensity ranges for RVS and SVS CR stimulation, separately. One could then select a RVS intensity close to the lower border of the RVS range and a SVS intensity that exceeds the RVS intensity, say, by 30% or is selected from the lower middle range of SVS intensities. These intensities could then be tested in a first man study of two-stage CR stimulation. Of course, based on the computational results obtained here, we cannot make any claims concerning safety, tolerability and efficacy in potential clinical applications.

## Author contributions

PT came up with the initial ideas for this work. MZ designed and performed the simulations. MZ and PT analyzed the data, discussed findings and interpretations, and wrote the manuscript.

### Conflict of interest statement

PT works at Jülich Research Center and as consulting professor at Stanford University. Several patents protect invasive and non-invasive CR neuromodulation. The main inventor of the CR patent portfolio is PT the assignee is Jülich Research Center. MZ works at Jülich Research Center. PT and MZ are co-inventors of recently filed two-stage CR patents.
